# The Effectiveness of Therapeutic Exercise Interventions With Virtual Reality on Balance and Walking Among Persons With Chronic Stroke: Systematic Review, Meta-Analysis, and Meta-Regression of Randomized Controlled Trials

**DOI:** 10.2196/59136

**Published:** 2024-12-02

**Authors:** Maria Krohn, Aki Rintala, Jaakko Immonen, Tuulikki Sjögren

**Affiliations:** 1 Faculty of Sport and Health Sciences University of Jyväskylä Jyväskylä Finland; 2 Physical Activity and Functional Capacity Research Group Faculty of Health Care and Social Services LAB University of Applied Sciences Lahti Finland; 3 Department of Medicine Wellbeing Services County of Central Finland Jyväskylä Finland

**Keywords:** stroke, chronic, virtual reality, physiotherapy, therapeutic exercise, balance, walking

## Abstract

**Background:**

Well-targeted balance, walking, and weight-shift training can improve balance capabilities in the chronic phase of stroke. There is an urgent need for a long-term approach to rehabilitation that extends beyond the acute and subacute phases, supporting participation without increasing the demand for health care staff.

**Objective:**

This study aims to evaluate the effectiveness of therapeutic exercise interventions with virtual reality (VR) training on balance and walking at the activity and participation levels in individuals with chronic stroke, compared with control groups receiving no treatment, conventional physical therapy, specific training, similar treatment, or identical treatment without VR.

**Methods:**

Studies were searched across 6 databases. The inclusion criteria were as follows: Adults aged 18 years or older with a stroke diagnosis for at least 6 months (population). Therapeutic exercises within a VR environment, using VR glasses or interactive games (intervention). Control groups without the use of VR (including no treatment, conventional physical therapy, specific training, similar treatment without VR, or identical treatment without the additional use of VR; comparison). We evaluated the Berg Balance Scale score, Functional Reach Test performance, Activities-specific Balance Confidence Scale score, Six-minute Walk Test, Two-minute Walk Test, 10-meter Walk Test results, and cadence (outcome measures). We investigated randomized controlled trials (study design). A meta-analysis and a meta-regression analysis were conducted to evaluate whether the content of VR interventions or control groups, as well as the level of VR immersion used, was related to balance or walking outcomes.

**Results:**

A total of 43 randomized controlled trials involving 1136 participants were included in this review. The use of VR training in therapeutic exercise interventions had a large effect on balance (standardized mean difference 0.51, 95% CI 0.29-0.72; P<.001) and a moderate effect on walking (standardized mean difference 0.31, 95% CI 0.09-0.53; P=.006) in individuals with chronic stroke, compared with pooled control groups (no treatment, conventional physical therapy, specific training, similar treatment, or identical treatment without the use of VR). According to the meta-regression findings, the content of VR interventions (P=.52), the type of control groups (P=.79), and the level of VR immersion (P=.82) were not significantly related to the pooled balance or walking outcomes. The GRADE (Grading of Recommendations, Assessment, Development, and Evaluations) was moderate for balance and low for walking.

**Conclusions:**

Therapeutic exercise training with VR had a positive, albeit moderate, effect on balance and a low impact on walking at the level of activity (capacity), even in the chronic phase of stroke, without serious side effects. The results are applicable to working-aged stroke rehabilitees who are able to walk without assistance. Further research is needed with defined VR methods and outcomes that assess performance at the level of real-life participation.

## Introduction

A stroke can be classified as either an infarction or a hemorrhage in the brain [1]. Causes of ischemic stroke include emboli from the heart, artery-to-artery emboli, and small-vessel disease [2]. The most common causes of hemorrhagic stroke are hypertension, cerebral amyloid angiopathy, and anticoagulation [3]. Stroke incidence, case fatality, and mortality rates vary even among countries with similar demographic and socioeconomic conditions [4]. Globally, age-adjusted stroke incidence ranges from 76 to 119 per 100,000 population per year [4]. Although age-adjusted stroke mortality has decreased worldwide, the absolute number of stroke survivors and the overall burden of stroke—including incidence, prevalence, deaths, and disability-adjusted life years—remain high and have increased over the years in low- and middle-income countries [5]. Therefore, there is an increasing need to develop therapeutic exercise interventions in physiotherapy (hereafter referred to as “therapeutic exercises”) that incorporate task-specific physical training without requiring an increase in health care staffing [6].

Rehabilitation research should place greater emphasis on individuals with chronic stroke. Increasing evidence shows that people with chronic stroke have slower walking speeds and reduced walking endurance compared with the healthy older age adult population [7]. Walking activity is lowest among individuals with stroke who experience the greatest balance impairments, highlighting the need to study the often-neglected chronic stroke population separately [8]. Research highlights the need for a long-term perspective on rehabilitation that extends beyond the acute and subacute phases to support participation in social and leisure activities [9]. Well-targeted balance, walking, and weight-shift training have been shown to improve balance capacities even in the chronic phase [10]. Incorporating virtual reality (VR) into physiotherapy could be an effective solution to enhance, maintain, and promote functional capacity and performance among individuals with chronic stroke. The advantage of VR is that the user feels present and interacts in real time with the virtual environment [11-13]. The degree of immersion in VR primarily depends on how much the user is surrounded by the virtual environment [14-16]. The highest level of immersion is achieved with head-mounted displays (HMDs), while moderate immersion uses wide curved or projection screens. Low immersion relies on traditional monitors or televisions, resulting in a significantly weaker sensory connection [17-20]. In all levels of immersion, the experience can be enhanced using a combination of visual, auditory, tactile, and motion-sensory inputs [16,21].

In recent decades, technology-assisted interventions have demonstrated benefits in stroke rehabilitation [22-24]. For example, a systematic review and meta-analysis by Cheok et al [22] reported that Nintendo Wii training, when combined with conventional rehabilitation, led to improvements in functional mobility for individuals with chronic stroke. However, other systematic reviews in the field have focused on all stages of stroke (ie, acute, subacute, chronic) without specifically targeting chronic stroke alone [22,23,25-31]. For chronic stroke, previous systematic reviews have primarily explored the effectiveness of VR interventions, focusing on outcomes such as walking [28], upper limb function and activity [6,32], or VR methods, without conducting meta-analysis or meta-regression [25,27]. Only a few meta-analyses have focused on chronic stroke, reporting the benefits of VR interventions on balance and physical function [33,34]. Additionally, a meta-regression indicated that the duration and frequency of VR training were not associated with its effects on physical function [34].

According to previous systematic reviews, the effectiveness of VR training as part of therapeutic exercise treatment has not been extensively studied in individuals with chronic stroke, and prior studies have often included various stages (acute, subacute, chronic) of stroke and different age groups in their analyses [23-34]. Additionally, small sample sizes, heterogeneity, and insufficient statistical power have been noted in these reviews, which lower the level of evidence [23,24,29,33]. There is a lack of information on balance and walking outcomes at the levels of activity and participation according to the International Classification of Functioning, Disability, and Health (ICF) framework [27,31,35]. A deeper understanding of how intervention-related factors influence rehabilitation outcomes is needed [33,34]. VR training in therapeutic exercise may offer new ways to practice movements in environments that would be more time-consuming and physically demanding in real life [36].

This systematic review with meta-analysis aims to investigate the effectiveness of VR as part of therapeutic exercise interventions on balance and walking at the ICF levels of activity and participation in individuals with chronic stroke. We addressed the following research question: What is the effectiveness of therapeutic exercise interventions incorporating VR training on balance and walking at the levels of activity and participation, compared with control groups receiving no treatment, conventional physical therapy, specific training, similar treatment, or identical treatment without the use of VR? In addition, meta-regression was used to investigate the components of interventions in both experimental and control groups, as well as balance and walking outcomes, and the effects of 3 levels of immersion in VR.

## Methods

### Identification and Selection of Studies

The protocol for our systematic review was published in PROSPERO’s International Registry of Impact Research (CRD42020184572) [37]. The search strategy was developed by members of the research team (TS, AR, Benjamin Waller, and Arja Piirainen). Specific search terms for each database were created in collaboration with a librarian (Anitta Pälvimäki) from the university’s health science department. The original search strategy is available in Multimedia Appendix 1. Searches were conducted using the following 6 databases: the National Library of Medicine’s Database (Medline), the Cumulative Index to Nursing and Allied Health Literature (CINAHL), the Excerpta Medica Database (Embase), the Cochrane Database of Systematic Reviews, the Physiotherapy Evidence Database (PEDro), and the Cochrane Controlled Trials Register (CENTRAL). The original systematic literature search covered studies published between January 2000 and May 2017. An updated search was conducted for studies published between June 2017 and September 2023. In accordance with the PRISMA (Preferred Reporting Items for Systematic Reviews and Meta-Analyses; Multimedia Appendix 2 [38]) guidelines [39], 2 reviewers (Benjamin Waller and Matti Munukka) independently screened all titles and abstracts of the studies using the Covidence program [40]. After the title and abstract screening phase, potentially relevant studies were independently evaluated for full-text assessment by pairs within the research team (Benjamin Waller, Matti Munukka, Susanne Aalto, Heidi Niemi, Heidi Nousiainen, or MK). The update was conducted by 2 researchers (MK and TS). In case of disagreement, a third reviewer (AR) was consulted. Additionally, a supplementary manual search was conducted on the accepted studies using their reference lists.

**Figure 1:**
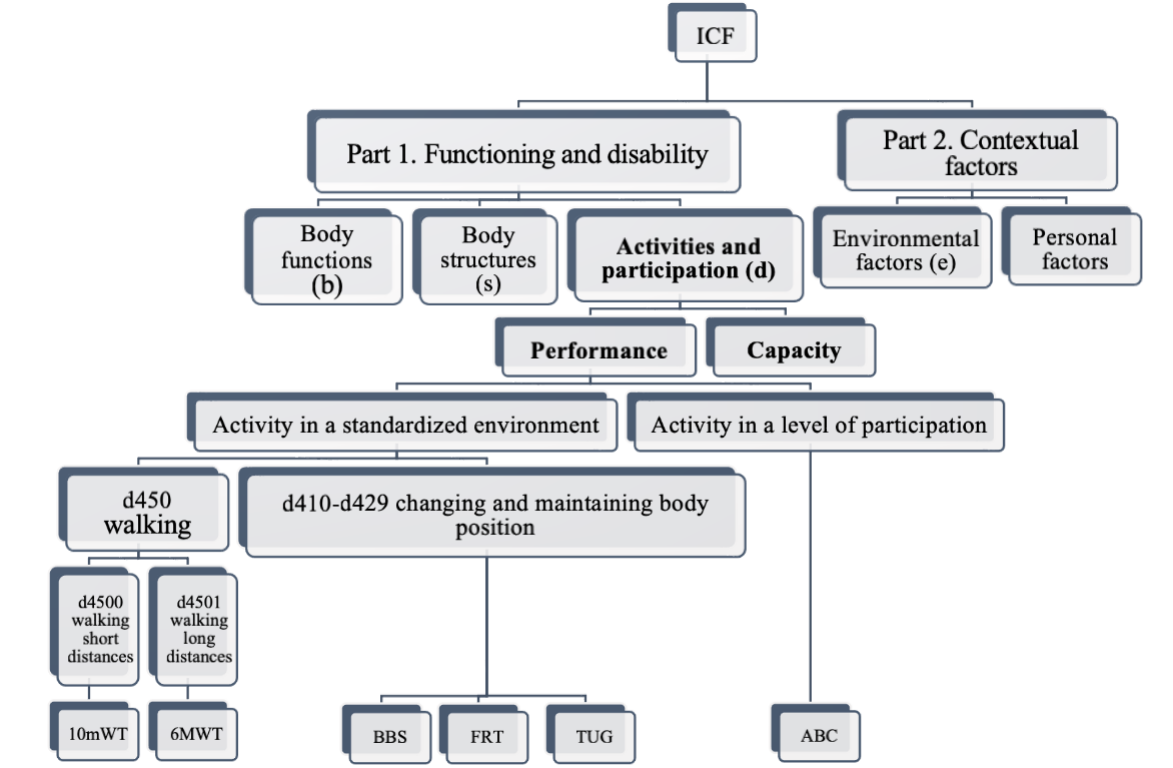
Categorization of walking, changing, and maintaining body position according to the ICF.

The PICOS (Population, Intervention, Comparison, Outcomes, Study) strategy [41] was used to define the research topic, formulate the research question, and establish the inclusion/exclusion criteria. All adults (over 18 years of age) with a stroke diagnosis for more than 6 months were included as the study population. The chronic stage of stroke is generally defined as more than 24 weeks (6 months) poststroke [42-44]. Research articles that did not clearly define cerebrovascular chronicity were excluded from the meta-analysis. Studies involving individuals with stroke identified as acute or subacute were also excluded (*population*). The *intervention* considered was therapeutic exercise (physiotherapy) sessions in a VR environment, using VR glasses or interactive games. We defined therapeutic exercise interventions as any movements systematically performed to enhance patients’ activity and participation or to prevent impairments, with the aim of reducing risks, optimizing health, and reinforcing fitness and well-being [45]. A list of accepted interventions was created before study selection (MK and TS). The definition of VR adhered to the principle established by Schultheis and Rizzo [46]: “An advanced form of human-computer interface that allows the user to interact naturally with a computer-assisted environment.” In addition to VR glasses, VR can be created through projection on a wall or through interactive gamification. The highest degree of immersion is experienced with various HMDs. Moderate immersion in VR is achieved using a wide curved screen [17] or by projecting screens onto walls [18]. In low immersion, the technology is typically limited to the use of a traditional monitor or television screen, resulting in a significantly weaker sensory connection compared with high-immersion experiences [19,20]. In all degrees of immersion, sensory experiences can be enhanced using a variety of visual, auditory, tactile, and motion stimuli [16-21]. For *comparison*, we chose no treatment, conventional physical therapy, specific training, similar treatment without VR, or identical treatment without the additional use of VR, pooled into a control group consisting of the aforementioned 5 control groups. The *outcomes* included balance or walking outcomes (see Multimedia Appendix 3 for the priority listing of accepted outcomes) at the ICF levels of activity (capacity) and participation (performance) (see Figure 1 for the categorization of walking, changing, and maintaining body position according to the ICF) [35,47]. Only originally published randomized controlled trials (RCTs) written in English were accepted (*study type*).

### Data Extraction

First, 2 members of the research team (Matti Munukka and Benjamin Waller) independently evaluated studies published between January 2008 and May 2017 based on the inclusion criteria using the Covidence program (Covidence 2020). In case of disagreement between the 2 screeners, a third reviewer (TS) resolved the issue by evaluating the study in question. Second, the updated search (January 2017-December 2019) was screened by 2 reviewers (MK and TS), with results confirmed by another member of the research team (AR). In addition, a manual search was conducted in September 2023. The research team (MK, AR, JI, and TS) collaboratively carried out the data extraction from the included RCT studies, which included details such as the main author, country, year of publication, population characteristics (eg, age, gender, affected side, and type of cerebrovascular accident), study duration, description of the VR intervention, description of the comparison, therapy frequency, measures used, and the main results.

### Risk of Bias

The methodological quality of the included articles was evaluated using the Cochrane Risk of Bias (RoB) tool [48]. The risk of bias was assessed by 1 researcher (MK) following an agreed-upon strategy among the research team. In case of disagreement, the consensus was reached by consulting a third reviewer (Susanne Aalto, Matti Munukka, Heidi Niemi, Heidi Nousiainen, Benjamin Waller, or TS). During data updates, the risk of bias assessment was conducted by 2 independent researchers (MK and TS). In case of disagreement, a third reviewer (AR) was consulted. Each article was categorized into groups of low, high, or unclear risk of bias based on the following criteria: random sequence generation (selection bias), allocation concealment (selection bias), blinding of participants (performance bias), blinding of personnel (performance bias), blinding of outcome assessment (detection bias), incomplete outcome data (attrition bias), selective reporting (reporting bias), and other sources of bias (eg, whether intervention and control groups were comparable in terms of demographic information or baseline measures). A high-quality study had to meet 5 weighted rating criteria, and the remaining points could not carry a high risk of error. The weighted rating domains included the randomization method, allocation concealment, blinding of evaluators, description of dropouts, and other potential sources of bias (eg, differences between experimental and control groups at baseline). A valid study required at least four low-risk ratings on the weighted criteria, and no valid study could have high-risk ratings on the other criteria.

### Statistical Analysis

Meta-analyses were performed separately for balance and walking outcomes using a predefined list of measurements for both outcomes (see Multimedia Appendix 3 for the priority listing of accepted outcomes). Studies that did not report adequate posttreatment values (mean and SD, or mean change and SD) were excluded from the meta-analysis. All meta-analyses were conducted using a random-effects model with standardized mean differences (SMDs). The SMD between the groups was classified as large (>0.5), moderate (0.3-0.5), small (0.1-0.2), or insubstantial (<0.1) [49]. Pooled effect estimates, representing a combination of single effects from the RCTs, were analyzed using the Review Manager 5.3 statistical software (Cochrane Collaboration) [50]. The percentage of variation across studies due to statistical heterogeneity, rather than chance, was assessed using the *I*^2^ statistic [51]. A percentage value close to 0 indicates a low level of variation across the studies [51]. The level of variation due to statistical heterogeneity was defined at the following thresholds: <40% was considered low, 30%-60% moderate, 50%-90% substantial, and >75% considerable [51]. Potential publication bias was evaluated using funnel plots [52] from the main analysis.

Sensitivity analyses were performed for both outcomes. The inclusion criteria for the sensitivity analysis were based on the Cochrane Risk of Bias tool [48]. Studies were excluded from the sensitivity analysis if they were pilot studies or studies had clearly inconsistent reporting of results.

Meta-regression, based on meta-analytic and linear regression principles, was used to explore the heterogeneity of the meta-analysis. The use of meta-regression offers better opportunities to investigate the clinical and methodological diversity, as well as the statistical heterogeneity often observed in meta-analyses [53]. The aim was to determine whether any statistical heterogeneity could be explained by performing analysis of variance–like analyses based on 4 categorical covariates. The first covariate related to the content of the experimental intervention with VR was as follows: 1=VR training alone; 2=specific physiotherapy (eg, progressive balance training or treadmill training) with VR training as an addition to that specific physiotherapy; and 3=nonspecific conventional physiotherapy with VR training as an addition. The second covariate related to the content of the control groups without VR was as follows: 1=no training, no physiotherapy, or placebo training; 2=conventional physiotherapy (traditional); 3=specific physiotherapy training, which was not comparable to the content of the experimental group; 4=comparable physiotherapy training in terms of content and duration; and 5=identical treatment as in the experimental group, but without the additional use of VR. The third covariate related to the degree of VR immersion was categorized as follows: low=monitor or television screen; medium=wide curved screen; and high=HMDs. The fourth covariate was the response outcome variables themselves: balance and walking. Three categorical variables were considered: control, intervention, and outcome variable (balance/walking). Each of these variables was fitted separately. R software (R Foundation) [54], specifically the “metafor” package [55], was used to fit the models. The analysis of categorical variables (in this case, balance and walking) is a special case of a linear model, which indicates level differences.

### Grading of Recommendations, Assessment, Development, and Evaluations (GRADE)

GRADE (Grading of Recommendations, Assessment, Development, and Evaluations) is a framework used to rate the certainty of evidence. The health benefits and harms of therapy are crucial when developing new guidelines. The GRADE approach categorizes the quality of evidence into 4 levels: high, moderate, low, and very low: “We are very confident that the true effect lies close to that of the estimate of the effect” (*high*); “We are moderately confident in the effect estimate. The true effect is likely to be close to the estimate of the effect, but there is a possibility that it is substantially different” (*moderate*); “Our confidence in the effect estimate is limited. The true effect may be substantially different from the estimate of the effect.” (*low*), and “We have very little confidence in the effect estimate: The true effect is likely to be substantially different from the estimate of effect.” (*very low*). The following factors can reduce the quality of evidence: limitations in study design or execution, inconsistency of results, indirectness of evidence, imprecision, and publication bias. However, a large effect size, dose-response gradient, and the impact of plausible residual confounding can increase the quality of evidence [56]. Triangulation by 3 researchers (MK, TS, and AR) was used to assess the quality of the evidence.

The quality of the RCT studies was evaluated using the kappa coefficient, which indicates the consistency of evaluation between 2 independent researchers (MK and TS). The consistency of categorization was classified as follows: over 0.75 indicates excellent agreement, 0.40-0.75 indicates fair to good agreement, and below 0.40 indicates poor agreement [57].

## Results

### Study Selection Process and Detailed Study Information

**Figure 2:**
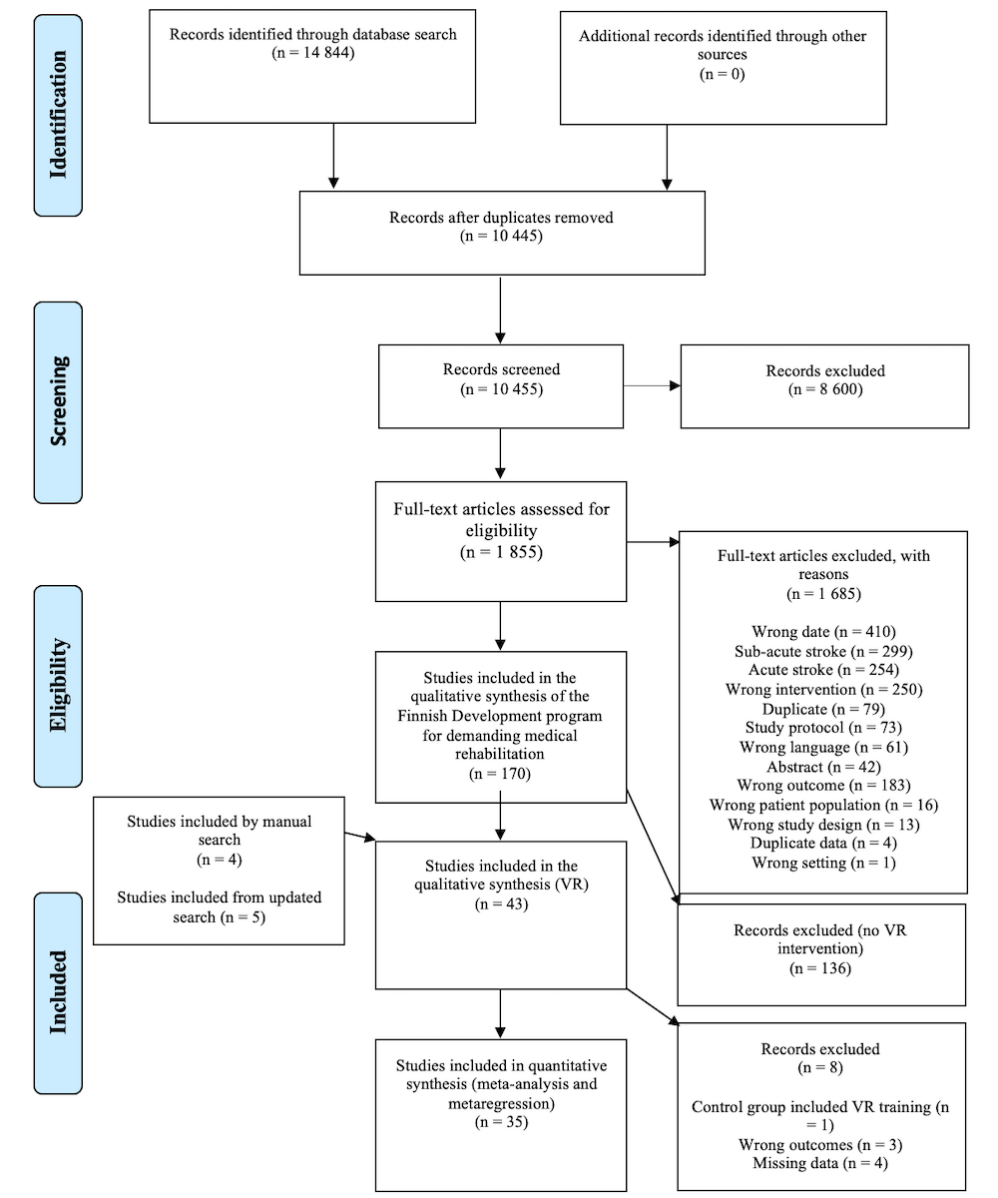
Flowchart of study selection.

The flowchart of study selection is presented in Figure 2. Detailed information on the interventions, comparisons, outcome variables, and inclusion criteria for the accepted studies is provided in Multimedia Appendix 4 (see also [49,58-99]).

### Description of the Participants

A total of 43 RCT studies (1136 participants) were included in this review, with VR utilized independently in 16 studies (246 participants) or as part of the therapeutic exercise intervention in 27 studies (343 participants). More than half of the participants were male, with an average age of 59 (SD 10.5) years. On average, stroke was diagnosed 31.1 (SD 11.9) months ago in participants. Based on the mean of 13 included studies, 214 out of 333 (64.3%) participants experienced ischemic cerebrovascular events, while 119 out of 333 (35.7%) experienced hemorrhagic events. Ten studies reported the type of cerebrovascular event, with infarction occurring in 176 of 282 (62.4%) cases and hemorrhage in 106 out of 282 (37.6%) cases. Twenty research articles did not specify the classification of the cerebrovascular accident; 454 of 862 (52.7%) participants were affected on the right side of the body, while 11 studies did not report the affected side.

The main inclusion criteria for the 43 RCT studies were adequate functioning in balance and walking ability (n=39, 91%), cognitive function and the ability to understand communication and instructions (n=36, 84%), as well as sight, hearing, and vestibular function (n=14, 33%). Additionally, participants were required to have no severe comorbidities (n=12, 28%) that could affect the therapeutic exercise. Cognitive function was generally defined as normal (Mini-Mental State Examination [MMSE] ≥24 points) [58-74] or as mild cognitive impairment (MMSE 19-23 points) [75-81]. In terms of physical functioning, there was more variation. Independent balance was mentioned in 14 studies (33%). Ten studies used a time-bound minimum requirement for standing position (minimum 30 seconds, maximum 30 minutes) [49,61,82-86], or sitting position (minimum 10 seconds, maximum 30 minutes) [49,71,87]. One study used the Berg Balance Scale definition (<56 sum score) [88]. In 3 studies, independent balance was not defined in more detail [79,92,95]. Independent walking was mentioned in 25 studies (58%). The minimum requirement was reported either in distance (10 or 15 m) [59,60,63,64,68-70,75,77,78,81,89,90] or in feet (10 feet=3.048 m) [82], or as a time-bound minimum (minimum 1 minute, maximum 30 minutes) [62,65,71,83,84,91]. Ten studies (23%) included both independent walkers and those with walking assistance [59,60,68,71,75,78,81,82,89,91]. More detailed inclusion and exclusion criteria of the original RCT studies are provided in Multimedia Appendix 4.

### Description of the Therapeutic Exercise Interventions and VR Technologies

In 13 studies [49,60,65,66,71,74,83,84,90-94], VR training was integrated with therapeutic exercises. The average duration of both therapeutic exercise and VR training was 5.6 (SD 2.4) weeks, with a frequency of 3.5 sessions per week. The average duration of each therapy session was 61.3 (SD 26.6) minutes. For VR training specifically, the average duration was 5.6 weeks (SD 2.4 weeks; range 3-12 weeks), with sessions occurring 3 times per week. The average duration of a VR session was 31.8 (SD 10.0) minutes. VR technologies varied across studies, and the specific VR technologies used are detailed in Multimedia Appendix 4. Dynamic balance training was the most common VR intervention, implemented in 25 of the 43 studies (58%) [60,61,63,68,69,72-74,78,80-88,90,92,93,95-98]. The second most common intervention was walking exercises, used in 11 studies (26%) [58,59,62,64-66,75,77,89,94,99], typically performed as treadmill exercises in a VR environment. In 9 studies [60,68,74,82,92,93,95,96,98], VR was implemented using the Nintendo Wii game console with a compatible balance board, which detects weight shifts and distributions. Four studies combined balance and stepping exercises [69,72,84,86]. Five interventions used the Nintendo Wii Sports with Wii Remote controllers, excluding the balance board [61,75,79,88,97], while 5 interventions utilized the Microsoft Kinect technology for VR training [72,75,81,82,85]. Additionally, other methods were used to create VR, including head-mounted VR glasses [49,66,70], the BioRescue platform [69], IREX (Immersive Rehabilitation and Exercise) technology [84], optic flow [77], real-world recordings [58,59], and the Logitech 29 unit with a monitor [67]. VR interventions provided real-time visual feedback on participants’ movements. In studies combining treadmill training with VR, the landscape change was adjusted to the participant’s walking speed. Most studies enhanced the VR experience with auditory recordings or game sounds.

The content of the experimental intervention with VR was classified as follows: VR training alone (n=14, 33%) [62,63,69,72,73,75,77,82,87-89,95,96,99], VR training as an addition to specific physiotherapy (n=2, 5%) [94,98], and nonspecific conventional physiotherapy with additional VR training (n=26, 60%) [49,58-61,64-68,70,71,74,76,78, 79,81,83-86,90-93,97].

The level of immersion of VR interventions used in the RCT studies was mostly low (n=30, 70%), with methods of moderate and high immersion used less frequently (n=12, 28%). The technology used included monitors or television screens in 30 studies [60,61,63,67-69,71,72,74-76,78-83,85,87-98], wide curved screens or projected screens in 9 studies [58,59,62,64,65,73,77,84,99], and various HMDs in 3 studies [49,66,70]. The degree of immersion could not be classified in 1 study [86].

### Description of the Interventions in Control Groups

Control groups mostly consisted of therapeutic exercises aimed at improving balance [59-61,63,68,74,80,81,83-88,90, 92,93,96-98] or walking [58,59,62,64,66,67,89,95,99] without VR technology. The content of the control intervention without VR was classified as follows: no training, no physiotherapy, or placebo training (3/43, 7%) [73,76,82]; conventional physiotherapy (8/43, 19%) [75,77,81,85,86,88,96,97]; specific physiotherapy training (8/43, 19%) [63,66-69,72,89,95], which was not comparable to the experimental group in terms of content and duration; and identical treatment to the control group (13/43, 30%) [49,65,70,71,74,79,83,84,90-94]. Conventional physiotherapy in the control groups typically involved progressive exercises using neurodevelopmental treatment, Bobath (the Bobath concept; neurodevelopmental treatment), and proprioceptive neuromuscular facilitation–based techniques. The control group generally received a combination of 2 or more treatments, such as a standard rehabilitation program and treadmill training. In 9 of 43 studies (21%) [58,59,62,64,66,67,89,95,99], the control groups received treadmill training (for a detailed description of the contents in control groups, see Multimedia Appendix 4).

### Description of the Used Outcome Measurements

Accepted outcome measures for balance included the BBS, Functional Reach Test (FRT), Timed “Up and Go” (TUG), and Activities-Specific Balance Confidence Scale (ABC). Among these balance measures, BBS, FRT, and TUG primarily assess short-term balance performance rather than participation, as they involve changing and maintaining body position in a controlled, standardized environment. The ABC was the only balance measure that assessed capacity, as it evaluates activity at the level of participation through questions about perceived confidence in various activities in nonstandardized environments. Walking under standardized conditions corresponds to the performance level of the ICF framework, while walking under changing (nonstandardized) conditions is related to the ICF level of participation [100]. See Figure 1 for the categorization of walking, changing, and maintaining body position according to the ICF. Accepted walking outcomes included the 10-meter Walk Test (10mWT), 2-minute Walk Test (2MWT), 6-minute Walk Test (6MWT), and cadence. All of these are performed in standardized environments, which means they do not capture potential changes at the level of participation. Because of the absence of outcomes that measure the level of participation, not all research questions can be addressed.

### Methodological Quality and the Risk of Bias

**Figure 3:**
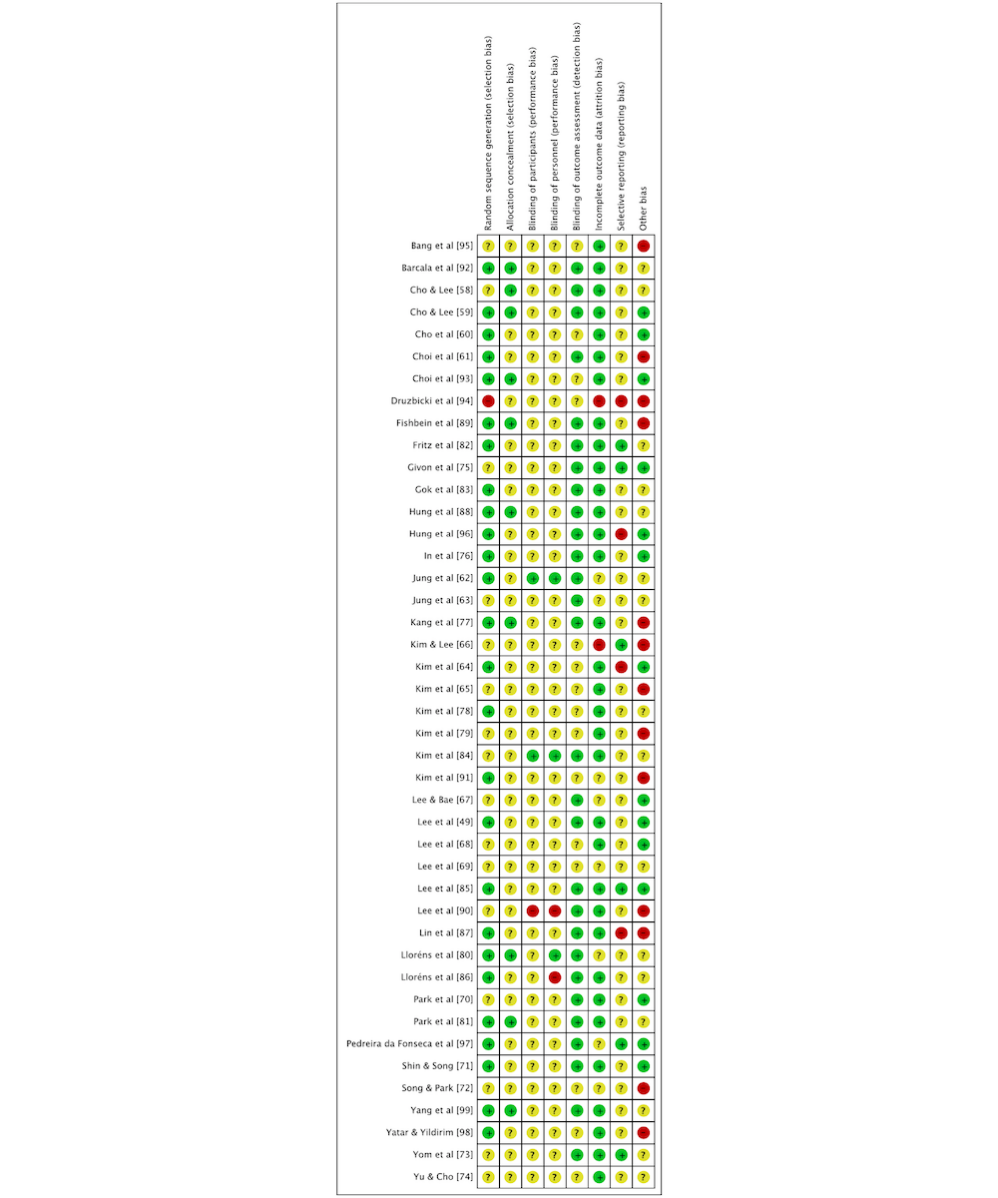
Risk of bias of the individual studies.

The overall methodological quality of the studies was unclear. As many as 17 out of the 43 studies were rated as having an unclear risk of bias [58,60,62,63,67-70,73-75,78,80,82-84,97]. One study demonstrated high-quality methodology [59], while 9 studies met acceptable quality standards [49,71,76,81,85,88,92,93,99]. The main methodological weaknesses contributing to the increased risk of bias were nonblinded treatment of participants, nonblinded caregivers, potential reporting bias, and insufficiently described randomization methods, which were not described in an acceptable manner. According to the quality assessment, the reliability of the meta-analysis and meta-regression was compromised due to an unclear risk of bias related to the blinding of participants and personnel in 36 studies (see Figure 3 for the risk of bias in individual studies; also see [49,58-99]). The kappa factor, which measures the consistency of the methodological quality evaluation between 2 independent researchers, was 0.64, indicating a good level of agreement [57].

### Effectiveness of VR Training on Balance: Meta-Analysis

**Figure 4:**
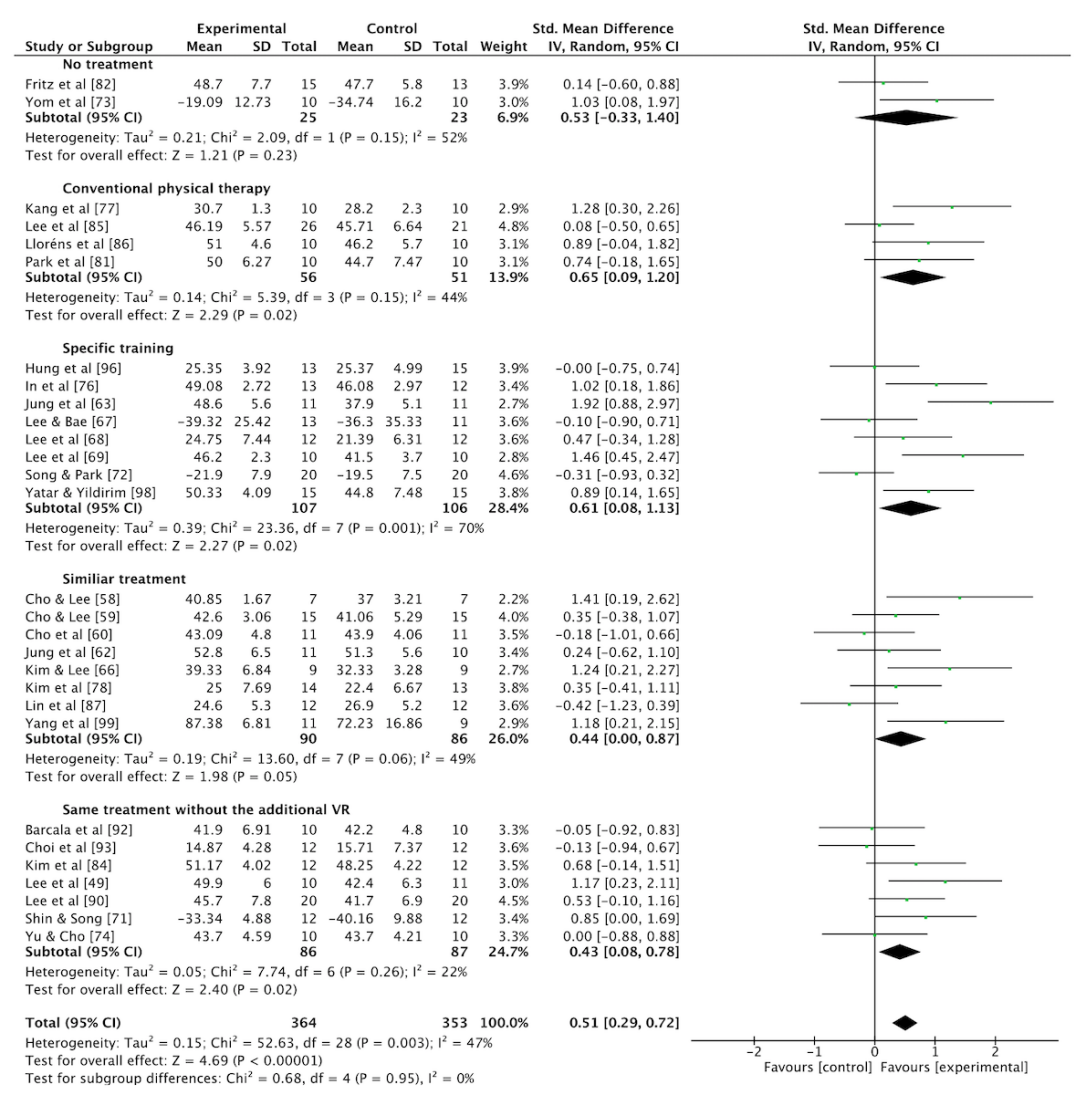
Standardized mean difference (SMD) in the effectiveness of VR interventions on balance compared to no treatment, conventional physical therapy, specific training, similar treatment, and same treatment without the use of VR.

A meta-analysis was conducted on 29 studies [49,58-60,62,63,66-69,71-74,76-78,81,82,84-87,90,92,93,96,98,99] (n=717) to assess balance outcomes. Therapeutic exercise interventions combined with VR training demonstrated a large effect on balance compared with control groups, which included no treatment, conventional physical therapy, specific training, similar treatment, or identical treatment without the use of VR (SMD 0.51, 95% CI 0.29-0.72; *P*<.001; Figure 4; also see [49,58-60,62,63,66-69,71-74,76-78,81,82,84-87,90,92,93,96,98,99]).

The results of the meta-analysis indicated moderate heterogeneity among the included studies (*I*^2^=47%). A funnel plot for the SMD analysis, which may suggest possible reporting bias, is presented in Multimedia Appendix 5.

In the sensitivity analysis, 2 studies were excluded: 1 due to being a pilot study [87] and the other because of inconsistent reporting regarding the TUG test results [72]. The sensitivity analysis of 27 studies [49,58-60,62,63,66-69,71,73,74, 76-78,81,82,84-86,90,92,93,96,98,99] (n=653) revealed that the statistically significant difference between the experimental and control groups remained (SMD 0.57, 95% CI 0.37 to 0.78; *P*<.001; Multimedia Appendix 6 (see also [49,58-60,62,63,66-69,71-74,76-78,81,82,84-87,90,92,93,96,98,99]). After the sensitivity test, the statistical heterogeneity, as measured by *I*^2^, was reduced to 37%.

### Effectiveness of VR Training on Walking: Meta-Analysis

**Figure 5:**
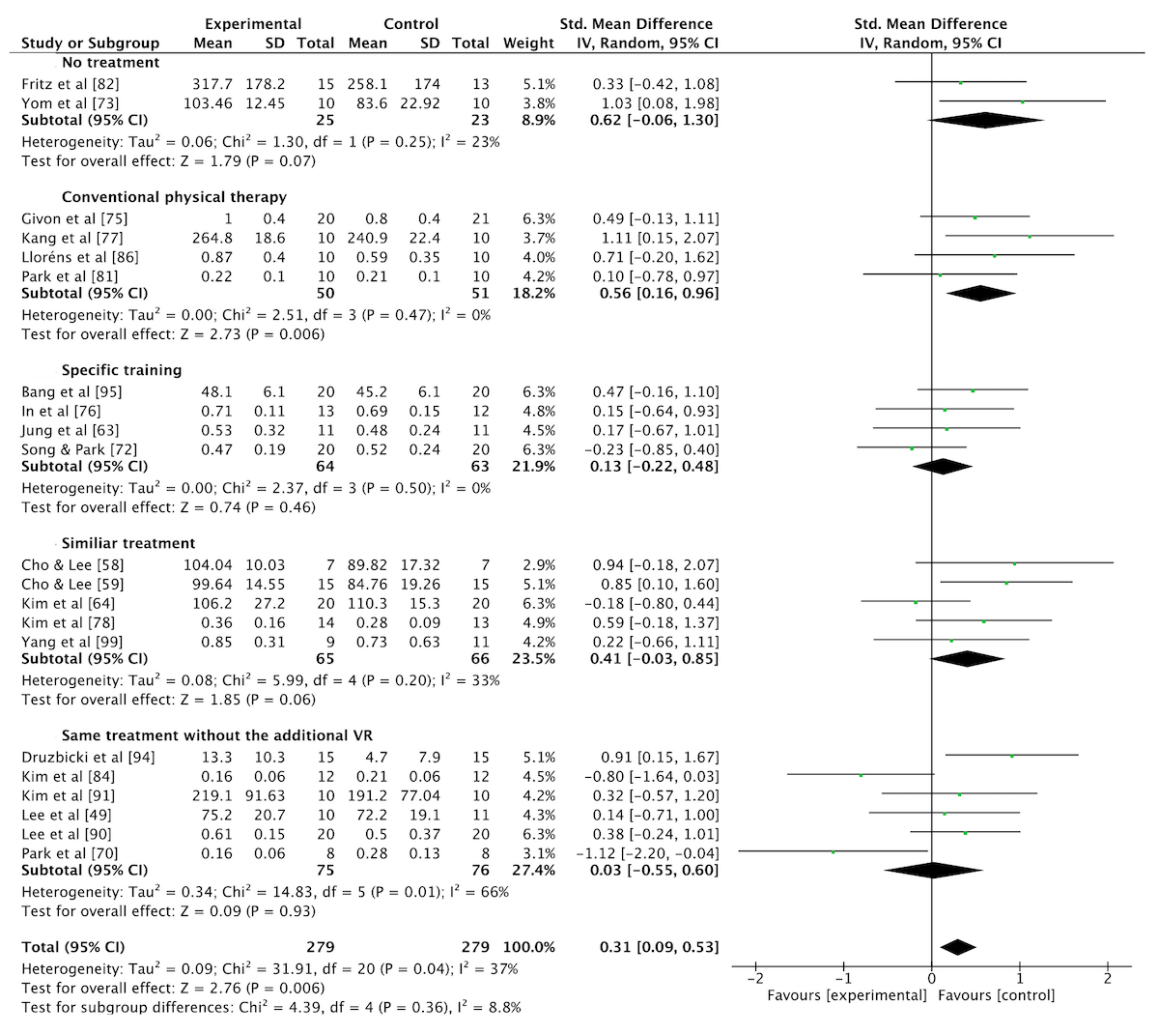
Standardized mean difference (SMD) of the effectiveness of VR interventions on walking compared to no treatment, conventional physical therapy, specific training, similiar treatment and same treatment without the additional VR.

A meta-analysis was conducted on 21 studies [49,58,59,63,64,70,72,73,75-78,81,82,84,86,90,91,94,95,99] (n=558) for walking outcomes. Therapeutic exercise interventions with VR training showed a moderate effect on walking compared with the pooled control group (which included no treatment, conventional physical therapy, specific training, similar treatment without VR, or identical treatment without the addition of VR) (SMD 0.31, 95% CI 0.09-0.53; *P*=.006; Figure 5; also see [49,58,59,63,64,70, 72,73,75-78,81,82,84,86,90,91,94,95,99]). A moderate level of statistical heterogeneity was observed (*I*^2^=37%). The funnel plot showing possible reporting bias is presented in Multimedia Appendix 5.

In a sensitivity analysis of walking outcomes (Multimedia Appendix 7; see also [49,58,59,63,64,70,72,73,75-78, 81,82,84,86,90,91,94,95,99]), 1 study was excluded due to inconsistent reporting of the 10mWT test results [72]. The sensitivity analysis of 20 studies [49,58,59,63,64, 70,73,75-78,81,82,84,86,90,91,94,95,99] (n=518) yielded similar results to the main analysis, with a slight increase in the SMD value (SMD 0.34, 95% CI 0.12-0.56; *P*=.002). The level of statistical heterogeneity remained moderate (*I*^2^=34%).

### Exploring the Methodological Diversity on Walking and Balance: Meta-Regression

A meta-regression was performed on 35 studies (n=938) for the pooled group of balance and walking outcomes. There were no statistically significant differences between intervention groups (*P*=.52), control groups (*P*=.79), degree of immersion (*P*=.82), or balance and walking outcomes (*P*=.74; see Table 1 for meta-regression covariates). The wide CIs suggest a large amount of uncertainty.

**Table 1:** Meta-regression covariates of therapeutic exercise interventions with VR^a^ on balance and walking among persons with chronic stroke.

Covariates			Estimate		SE		*Z* value		*P* value (95% CI)		Omnibus *P* value	
**Content of the experimental intervention**												.52
	VR alone (intercept)	0.449		0.163		2.751		N/A^b^				
	Specific physiotherapy + VR	0.453		0.420		1.079		.28 (–0.370 to 1.276)				
	Conventional physiotherapy + VR	–0.009		0.207		–0.045		.96 (–0.414 to 0.396)				
**Content of the control intervention without VR training**												.79
	No physiotherapy or training (intercept)	0.697		0.349		1.997		N/A				
	Conventional physiotherapy	–0.203		0.423		–0.480		.63 (–1.031 to 0.625)				
	Noncomparable specific physiotherapy	–0.079		0.417		–0.188		.85 (–0.895 to 0.738)				
	Comparable physiotherapy (content and time)	–0.409		0.403		–1.016		.31 (–1.198 to 0.380)				
	Similar physiotherapy	–0.232		0.399		–0.581		.56 (–1.013 to 0.550)				
**Degree of VR immersion**												.82
	Low; monitor or television screen (intercept)	0.425		0.119		3.563		N/A				
	Medium; wide curved screen	0.155		0.245		0.636		.52 (–0.324 to 0.635)				
	High; head-mounted displays	0.059		0.395		0.148		.88 (–0.715 to 0.832)				
**Response outcome**												N/A
	Balance (intercept)	0.493		0.116		4.257		N/A				
	Walking	–0.072		0.217		–0.330		.74 (–0.496 to 0.353)				

^a^VR: virtual reality.

^b^N/A: not applicable.

### Strength of Research Evidence According to GRADE Evaluation

GRADE evaluation was conducted using the results from the meta-analysis and descriptive analyses (see Table 2 for factors contributing to the grade of evidence). The GRADE evaluation was downgraded for balance outcomes from high to moderate and for walking outcomes from moderate to low, primarily due to the methodological quality of the studies (risk of bias), clinical heterogeneity (inconsistency), and the low number of participants included in the meta-analysis (imprecision). Similar observations were made for other outcomes, but these were based on descriptive analysis since meta-analyses could not be performed due to the lack of specific data.

**Table 2:** Grade of evidence of VR^a^ training in balance and walking among chronic stroke rehabilitees.^b,c,d^

Outcomes and number of studies	Risk of bias	Inconsistency	Indirectness	Imprecision	Publication bias	Quality of the evidence (GRADE^e^)	Comments
Balance outcomes (29 RCT^f^ studies)	Evaluated study quality using the Cochrane Risk of Bias tool (unclear in 14/29). The primary methodological faults that increased the risk of bias were nonblinded treatment of the patients, nonblinded caregivers, possible reporting bias, and randomization of the studies, which was not described in an acceptable manner.	Technology varied between Wii Fit, Wii Fit Balance Board, real-world video recordings, VR reflection therapy, optic flow, and Microsoft Kinect training. The control group received traditional physical therapy, no intervention, treadmill walking, weight-shift exercises, proprioceptive neuromuscular facilitation training, or progressive balance training. Moderate statistical heterogeneity (I2=47%).	Chronic stroke survivors (>6 months)	A meta-analysis of 29 studies with sample sizes ranging from 7 to 21 participants (n=717).	The funnel plot indicated possible reporting bias.	⊕⊕⊕ Therapeutic exercise interventions with VR training had a large effect on balance compared with control groups, which consisted of a combination of no treatment, conventional physical therapy, specific training, similar treatment, or identical treatment without the use of VR (standardized mean difference 0.51, 95% CI 0.29-0.72; *P*<.001).	Methodological quality indicated a somewhat risk of bias. Clinical heterogeneity was observed in the use of technology and in the treatments in control groups.
Walking outcomes (21 RCTs)	Evaluated study quality using the Cochrane Risk of Bias tool (unclear in 9/21)	The technology used in the experimental groups was treadmill training in a VR environment. The control group consisted of moderate statistical heterogeneity (I2=37%).	Chronic stroke survivors (>6 months)	A meta-analysis of 21 studies with a sample size ranging from 7 to 21 participants (n=558).	Funnel plot indicated possible reporting bias.	⊕⊕ Therapeutic exercise interventions with VR training had a moderate effect on walking compared with the control group consisting of no treatment, conventional physical therapy, specific training, similar treatment with no VR, or identical treatment without the additional use of VR (standardized mean difference 0.31, 95% CI 0.09-0.53; *P*=.006).	Clinical heterogeneity observed in the compared treatments of control groups.

^a^VR: virtual reality.

^b^Patient or population: persons with chronic stroke receiving VR rehabilitation.

^c^Settings: home or rehabilitation care facilities.

^d^Intervention: VR-based therapeutic exercise.

^e^GRADE: Grading of Recommendations, Assessment, Development, and Evaluations

^f^RCT: randomized controlled trial.

## Discussion

### Principal Findings

The results of this review provide updated insights into the benefits of VR training on balance and walking, viewed through the ICF framework, and enhance understanding of the effectiveness of therapeutic exercises incorporating VR. Our meta-analysis revealed a statistically significant large effect on balance within the ICF domains of activity and participation, and a moderate effect on walking, for therapeutic exercise interventions that included VR training compared with the pooled control group (which consisted of no treatment, conventional physical therapy, specific training, similar treatment, or identical treatment without the use of VR). The findings confirm the small [24] to moderate [32] effects on lower limb functioning and overall functioning. At the same time, our results highlight the challenges identified by previous systematic reviews on VR-assisted physiotherapy: small sample sizes, heterogeneity, insufficient statistical power, and low levels of evidence [24,33,34]. Furthermore, clinical importance [33] or the quality of evidence (eg, GRADE) [34] has not been adequately addressed in previous reviews.

Compared with previous reviews [33,34], our review provides a deeper focus on the outcomes of balance and walking within the ICF components of activities and participation [35]. Additionally, we attempted to investigate the heterogeneity associated with balance and walking outcomes by examining several covariates. According to our findings, neither the content of VR interventions nor the level of VR immersion used in the interventions was significantly related to the outcomes of balance or walking. Furthermore, the outcome variables used (balance and walking) were not associated with the heterogeneity of the results. Previous systematic reviews with meta-analysis and meta-regression have observed that the heterogeneity in VR intervention duration or weekly frequency was not significantly related to physical function outcomes [34].

This review of the used interventions (covariates 1-3) and outcome-based covariates (covariate 4) was insufficient to investigate the heterogeneity of results, as no associations were found between the covariates and pooled outcomes (balance and walking). Potential reasons for the lack of significant associations between the covariates may include the homogeneity of the research material and interventions. First, the selected RCT studies’ inclusion and exclusion criteria focused on normal cognitive function and adequate physical functioning, with an emphasis on independent walking, with or without assistive devices. The only clear exception was the study by Lee et al [49], in which cognitive function was defined as below normal on the MMSE scale (<24). Second, the differences in the contents of the experimental and control groups, both within and between groups, were relatively minor. The contents of the experimental interventions in groups 1-3, as well as the control interventions in groups 2-5, are largely based on the basic principles and practices of physiotherapy science (eg, Core Competence of Physiotherapy 2016 [101]). The only exceptions were 3 control groups, where the content consisted of no treatment, including no training, no physiotherapy, or placebo training (n=3) [73,76,82]. However, the control group with no treatment was underrepresented (3/43, 7%) in this review. Third, a low level of immersion in VR was most commonly used (n=30, 70%), while HMDs, which were classified as a high level of immersion, were rarely used [49,66,70]. It is worth noting that a study-level meta-analysis does not allow for examining individual-level relationships between effect size and covariates. Study-level associations are generally weaker, making them harder to identify.

Alternative covariates that could be tested in future research on balance and walking are biopsychosocial functioning status at baseline, support for human dignity and a sense of meaningful life in therapeutic exercise with VR, VR technology as an enabling tool for motivation and commitment, dose-response relationships related to therapeutic exercise training and physiotherapist support, and broader intervention content according to the ICF framework (functioning at the level of the body, the individual, and as a member of society). The use of more detailed covariates requires careful planning, implementation, and reporting in VR RCT studies. The level of reporting in the RCTs selected for this systematic review is not yet sufficient to incorporate these covariates into a more detailed analysis of the study impact (eg, using them as covariates in meta-regression).

Meta-regression is a sophisticated tool used to explore heterogeneity by examining whether a linear relationship exists between selected outcome measures and 1 or more covariates. However, associations found in a meta-regression should be considered hypothesis-generating rather than evidence of causality. Many clinicians, such as physiotherapists, as well as policy decision makers in physiotherapy and rehabilitation, may still be unfamiliar with the principles and assumptions underlying meta-regression [53].

In the following paragraphs, we discuss the results related to balance and walking in greater detail, in relation to the findings from previous systematic literature reviews.

### Effectiveness of VR Interventions on Balance in Persons With Chronic Stroke

Our study focused on balance outcomes because poor dynamic balance is strongly associated with an increased risk of falls in patients with stroke, which can hinder participation in social activities [102]. Dynamic balance tests, such as TUG and BBS, are often better predictors of falls compared with static balance tests [102]. Our meta-analysis demonstrated a statistically significant effect on balance in therapeutic exercise interventions with VR training compared with the pooled control group, which included no treatment, conventional physical therapy, specific training, similar treatment, or identical treatment without the use of VR.

Our study provides updated and more detailed knowledge on the effects of therapeutic exercises on balance in individuals with chronic stroke, as our systematic review includes 7 newly published RCTs [61,71,74-76,93,95] that were not part of the latest systematic literature review by Iruthayarajah et al [33]. Our findings align with those of Iruthayarajah et al [33], where the authors reported significantly higher BBS scores in the VR balance training group (including Nintendo Wii Fit Balance Board training, VR combined with treadmill training, or postural VR training) compared with the control group, which received alternative rehabilitation therapy [33].

In addition, using the ICF as a framework revealed a lack of outcomes related to participation (performance) in previous RCTs on physiotherapy (see Multimedia Appendix 4 for outcome variables of the included studies). As a result, the research question concerning balance and walking outcomes can only be answered at the ICF performance level (activity in a standardized environment). This highlights the need for more research on the effects of VR training at the ICF participation level.

### Effectiveness of VR Interventions on Walking in Persons With Chronic Stroke

Our findings indicated a moderate effect on walking in therapeutic exercise interventions with VR training compared with control groups without the use of VR for individuals with chronic stroke. Our research design clearly differs from previous systematic literature reviews, offering new insights into the effectiveness of VR on walking in individuals with chronic stroke. In previous reviews examining the effects of VR on walking, no meta-analysis was performed combining walking outcomes or prioritizing long-distance walking [6,24,26,28]. Our study provides new insights into the effects of therapeutic exercises on walking in individuals with chronic stroke, as previous reviews focused on stroke populations with varying disease durations [6,24,26,28]. In the review by Rodrigues-Baroni et al [28], a significant difference was observed in favor of VR practitioners regarding walking, as assessed by comfortable gait speed, compared with a placebo or no intervention. Additionally, in contrast to other systematic literature reviews, our study evaluated walking using both short-distance walking tests (2MWT) and long-distance walking tests (6MWT). Our findings on walking outcomes can only be interpreted from the ICF performance level, as all tests were conducted in standardized environments, leaving capacity (activity at the level of participation) unexamined. In our study, therapeutic exercise interventions were not limited to walking exercises alone; therefore, the effects were less pronounced than those observed in the study by Rodrigues-Baroni et al [28], although the results remain consistent.

### Risk of Bias

In this review, the overall risk of bias in studies included in the meta-analysis was assessed using the RoB with weighted rating criteria. Of the 43 studies, the risk of bias was found to be low in 10 (23%), high in 16 (37%), and raised some concerns in 17 (40%) RCTs included. These findings are somewhat aligned with those of Hohenschurz-Schmidt et al [103], who reported a risk of bias of 17% low, 44% high, and 38% with some concerns, as assessed using the updated Cochrane RoB tool (RoB 2). The comparison article [103] focused on physical, psychological, and self-management interventions for pain, providing general insights into risk assessment levels in physiotherapy, rehabilitation, and self-care studies. It also highlights potential differences between the Cochrane Risk of Bias tools (RoB and RoB 2), suggesting the need for further investigation. Since 2024, it has been recommended that the Cochrane Risk of Bias tool RoB 2 be used for all randomized trials in Cochrane Reviews [104].

In our research, the weighted quality criteria included blinding of rehabilitees and therapists, which lowered the overall quality assessment. Blinding the assessor and reporting it within the study would be an effective way to reduce the risk of unclear bias. A common issue in the original RCTs included in our systematic review—and in therapeutic exercise studies in physiotherapy more broadly [103]—was the lack of blinding among participants and those delivering the interventions to the intervention groups. This challenge arises because participants play an active role during interventions. Future research should explore solutions to the difficulties of implementing blinding in physiotherapy studies or consider addressing the issue from the perspective of the control group. Hohenschurz-Schmidt et al [103] emphasized how control design features—such as the number of treatment sessions, application methods, individualization of interventions, patient participation, fidelity monitoring, and treatment environment—can all influence outcomes. Similarly, in our review, we evaluated the diversity of VR interventions by classifying the content of the experimental and control groups and assessing how these factors were related to balance or walking outcomes.

Commonly used practices, such as standardizing registration and preregistration of RCTs (eg, ClinicalTrials.gov or ISRCTN Registry) and systematic reviews (eg, PROSPERO), could enhance research reliability. Accurately reporting dropout rates and consistently presenting all outcome variable results could further improve the quality of research articles [105,106]. If the quality of the RCTs included in a systematic review is not clearly defined, it can undermine the credibility of the results, the generalizability of findings, and the clinical implications [107]. In this review, the presence of an unclear risk of bias in many studies reduced the level of research evidence according to the GRADE evaluation. To achieve greater internal reliability, VR RCT studies should be reported more clearly and systematically, with a more accurate evaluation of bias and the use of internationally standardized tools and methods. Additionally, new analytical approaches should be developed to account for the multidimensional factors related to rehabilitee functioning, as well as individual and environmental factors, which may contribute to result heterogeneity in meta-analyses [107].

### Study Strengths and Limitations

The phenomenon of VR training has not yet been sufficiently investigated, but there are reasons to believe that VR-assisted therapeutic exercise contributes to improving physical functioning (balance and walking) in individuals with chronic stroke. The strengths of our study include the careful planning of the systematic review (including review registration), accurate thematic classification of the content of the experimental and control groups, levels of VR immersion, the outcomes used, and sophisticated meta-regression analyses to explore heterogeneity and the consistency of results. Furthermore, we gained updated insights into balance and walking among chronic stroke rehabilitees and conducted a thorough GRADE evaluation.

One limitation, related to study selection bias, is that our search strategy was not extended to national and regional databases, reference indices, the Scopus database, Google Scholar, Microsoft Academic search engine, doctoral dissertations, and thesis databases, as well as gray literature, such as reports, conference summaries, and unpublished or nonbibliographic sources [108]. Other clear limitations of the study include shortcomings in the quality of the original RCTs, for which no clarification was sought from the authors, and the inability to control for heterogeneity in balance and walking outcomes. The intervention-related covariates we used did not provide additional insights into the associations related to the heterogeneity of balance and walking results. Additionally, an older version of the RoB was used to evaluate the original studies.

The unclear risk of bias in many studies highlights its potential impact on validity. This potential bias was exacerbated by the nonblinding of participants and caregivers to treatment, reporting biases, and inadequate or unclear randomization procedures. Considering the strengths and weaknesses of this systematic review’s results, the GRADE of evidence [56,109], and the unclear risk of bias in methodological validity, the evidence is downgraded to moderate for balance and low for walking.

### Future Recommendations

Qualitative studies on the experience of VR technology among individuals with chronic stroke should be recommended, as VR technology may alter the nature of communication between the therapist and the participant. For instance, Sjögren and Korpi [110] used thematic analysis and inductive synthesis of qualitative studies among stroke and multiple sclerosis rehabilitees to identify key elements of (digital) technology–assisted physiotherapy. These themes included support for motivation and commitment, enablement of social interactions and relationships, design of safe and variable training environments, flexibility in choosing relevant and meaningful activities, identification of rehabilitation needs and goals, and support for rehabilitees to understand their current functioning status and the appropriate paths to improvement [110].

We observed a lack of studies using VR technology to measure the level of participation according to the ICF. We argue that there is still insufficient evidence regarding the effectiveness of VR technology among chronic stroke populations, and changes in activity and participation are not clearly defined at the ICF levels. For example, the outcomes in this meta-analysis were primarily short performance tests conducted in a clinical setting, which may not necessarily reflect changes in activities of daily living functions in participants’ everyday environments.

The use of VR methods in a home environment as part of self-rehabilitation should be explored more specifically, as many VR methods provide real-time feedback on body movements, aiding in relearning. A comparative study of VR methods with standardized training volumes and durations should be conducted to determine which VR methods are most effective. To enable independent VR training in the home environment, VR should also be studied without the guidance of a physiotherapist or other health professionals. In such research designs, safety factors and careful selection of the appropriate target group should be prioritized.

In this review, we found that physiotherapy still primarily uses commercial devices that provide less immersion, rather than VR technology, which offers greater immersion (30/43, 70%). A similar trend has been observed in previous rehabilitation studies (eg, Saposnik et al [111] and Laver et al [6]). One reason for this may be the high cost, limited availability, or nausea associated with using more advanced VR headsets [112,113], as well as the fact that few devices have been specifically developed for physiotherapy and physical rehabilitation. To date, our research evidence does not show a statistically significant advantage for deeper immersion technologies compared with lower levels of immersion. However, commercial VR methods may still be useful as part of rehabilitation, increasing the overall amount of training, and they are more accessible to caregivers.

Lohse et al [31] criticized the small number of participants in RCT studies, as well as the insufficient determination of conventional therapy, adherence effects, and motivational components in the original studies. In addition, our research highlights the need for future studies to take a closer look at VR technology interventions from the perspective of physiotherapy, specifically examining what kind of functional capacity training prerequisites VR enables and promotes, and how this activity is connected to the rehabilitee’s meaningful everyday life. For example, it is important to deepen our understanding of how exercises with VR glasses or lower extremity platforms differ, taking into account biopsychosocial functioning, cognitive requirements, and the degree of immersion. Additionally, it is crucial to determine whether the observed changes have significance for the rehabilitee’s functioning, such as changes in body function, body structures, capacity, and performance.

To enhance knowledge and understanding, there is an urgent need for RCT studies that also consider functional performance across varying operating environments. The RCT studies included in our review primarily focused on balance or walking outcomes at the performance level. In the future, greater attention should be given to the quality of RCT research. It would also be important to more accurately investigate the potential connections between motivation and outcome variables. Rohrbach et al [114] also emphasized the need for standardized terminology and outcome measures to better understand how factors such as enjoyment, engagement, motivation, immersion, and presence contribute individually or interactively to VR intervention effectiveness. For example, rehabilitee motivation could not be used as a covariate, as none of the original studies included it as an outcome.

### Generalizability and Clinical Implications

Chronic stroke rehabilitation is a comprehensive field that includes participants with varying limitations and levels of function. Therefore, individuals with chronic stroke should not be viewed as a homogeneous group, but rather as persons with specific, individualized needs. In this review, the results cannot be generalized to the entire population, as the volunteers selected for the RCT studies do not fully represent the target group. The mean age of the participants included in our review (59 years) was significantly lower than the average age for men (69 years) and women (73 years) with their first cerebrovascular disease in Western Europe [115,116]. Additionally, based on the inclusion and exclusion criteria of our study, the participants in the selected RCTs had physical and cognitive functioning at a level that allowed them to engage in demanding balance and walking training with VR without assistance. Furthermore, the participants did not have severe comorbidities. These factors limit the applicability of our study results to older populations or rehabilitees with more significant functional impairments or multimorbidities. Future VR studies should include categorized subgroup analyses to provide more comprehensive clinical recommendations.

Despite the limitations previously mentioned, our findings may be useful for health care professionals, especially physiotherapists, working with individuals with chronic stroke. They could consider integrating VR technology into their clinical practice as an adjunct to conventional therapy to increase the amount of therapeutic activity.

In terms of clinical implications, it is important to consider not only the groups of rehabilitees in one’s practice but also potential adverse effects and factors related to defining clinically significant results. While our results indicated a large effect on balance and a moderate effect on walking, the clinical significance of these findings remains partly unclear. VR training did not improve BBS scores to a clinically significant level compared with rehabilitees who did not participate in VR training [117]. However, according to the review by Downs et al [118], a mean difference of more than 3 points may be considered clinically significant if the baseline scores are between 20 and 50 points.

This systematic review of the included VR studies reported some minor adverse effects. Park et al [70] noted dizziness caused by VR glasses and a time delay between real-time and reference motion, which resulted in visual feedback not occurring on schedule. Hung et al [96] reported a possible increase in spasticity, leading to a higher risk of falling as an adverse effect in their study. The authors highlighted that the safety of VR training could be improved by ensuring the physiotherapist and, if necessary, relatives are familiar with VR technology, as well as by providing a barrier-free and quiet training environment. They also argued that, for the most part, VR training related to balance and walking is not suitable for wheelchair users [96].

More research is needed with outcome measures at the participation level, such as long-distance walking or the ABC test. Original RCT studies utilizing deeper levels of immersion (eg, head-mouth devices) in the VR world are also required. For better implementation of the research findings, accurate definitions of chronic stroke rehabilitators’ entry levels are necessary, along with solutions for studying rehabilitees who are not able to walk independently without assistance. Considering the inclusion and exclusion criteria of the original studies, rehabilitees with limitations in vision, hearing, cognition, sense of touch, or physical function are often excluded. Current research settings tend to focus on chronic stroke rehabilitators with fewer disadvantages. This raises the question of whether VR can be effective if the baseline level is low.

### Conclusions

The use of VR training in therapeutic exercise interventions may improve balance and walking in persons with chronic stroke. However, this review suggests that no significant association was found between the content of VR interventions, the control group, or the level of immersion used on balance and walking outcomes. The evidence was of moderate certainty for balance and low certainty for walking.

For clinical implementation, statistically significant results were achieved on average with the following factors: VR training using commercially available technology in addition to conventional training, 3 times a week, with each session lasting 30 minutes. This approach was applied to individuals with chronic stroke who had sitting and standing balance, no assistive devices, and the ability to walk independently. High-quality RCTs are needed to evaluate VR interventions for chronic stroke, specifically focusing on balance and walking at the ICF participation level. Future studies should also include participants of varying ages, functional abilities, and comorbidities to help generalize the results to a broader population of individuals with chronic stroke.

## Appendix

Multimedia Appendix 1 The original search strategy.

Multimedia Appendix 2 PRISMA 2020 checklist.

Multimedia Appendix 3 Prioritization of balance and walking outcomes in the meta-analysis and meta regression.

Multimedia Appendix 4 Description of interventions, comparisons, outcome variables and inclusion criteria of included studies.

Multimedia Appendix 5 Funnel plot of standardized mean difference (SMD) analysis with balance and walking outcomes.

Multimedia Appendix 6 Sensitivity test for standardized mean difference (SMD) of the effectiveness of therapeutic exercise interventions utilizing VR technology on balance.

Multimedia Appendix 7 Sensitivity test for standardized mean difference (SMD) of the effectiveness of therapeutic exercise interventions on walking utilizing VR technology.

## Abbreviations

10mWT: 10-meter Walk Test
2MWT: 2-minute Walk Test
6MWT: 6-minute Walk Test
ABC: Activities-Specific Balance Confidence Scale
BBS: Berg Balance Scale
CENTRAL: Cochrane Controlled Trials Register
CINAHL: Cumulative Index to Nursing and Allied Health Literature
Embase: Excerpta Medica Database
FRT: Functional Reach Test
GRADE: Grading of Recommendations, Assessment, Development, and Evaluations
HMD: head-mounted display
ICF: International Classification of Functioning, Disability, and Health
MMSE: Mini-Mental State Examination
PEDro: Physiotherapy Evidence Database
PICOS: Population, Intervention, Comparison, Outcomes, Study
PRISMA: Preferred Reporting Items for Systematic Reviews and Meta-Analyses
RCT: randomized controlled trial
SMD: standardized mean difference
TUG: Timed “Up and Go”
VR: virtual reality

## Data Availability

The data generated during or analyzed during this study are available from the corresponding author upon reasonable request.
