# Supplementary material for: The Effectiveness of Therapeutic Exercise Interventions With Virtual Reality on Balance and Walking Among Persons With Chronic Stroke: Systematic Review, Meta-Analysis, and Meta-Regression of Randomized Controlled Trials
Source: J Med Internet Res. 2024 Dec 2;26:e59136. doi: 10.2196/59136 (PMC11650088; doi:10.2196/59136)
Supplement: Multimedia Appendix 1 [file jmir_v26i1e59136_app1.docx]

**Database: Ovid MEDLINE(R) <1966 to May 2017>**

**Search Strategy:**

1. (stroke* or poststroke* or cvi or cva or apoplex*).mp.

2. (hemiparesis or hemiplegia or paresis).mp.

3. ((brain or cerebr* or cerebell* or intercran* or intercerebral or cerebrovasc*) and (disease* or accident* or haematoma* or hematoma* or infarct* or bleed* or disorder* or ischemi* or ischaemi*)).mp.

4. 1 or 2 or 3

5. exp exercise therapy/ or exp physical therapy modalities/

6. (physical therap* or physiotherap* or functional therap* or occupational therap* or rehabilitation or exercise* or training).mp.

7. physical fitness/ or "physical and rehabilitation medicine"/ or "physical therapy modalities"/ or exercise*/ or "stroke rehabilitation"/

8. (movement*/ or function* or aquat*) AND (terap*/ or exercise*/ or treatment*/ or training)

8. 5 or 6 or 7

9. Randomized Controlled Trials as Topic/

10. Randomized Controlled Trial/

11. Random Allocation/

12. Double-Blind Method/

13. Single-Blind Method/

14. Clinical Trial/

15. clinical trial, phase i.pt.

16. clinical trial, phase ii.pt.

17. clinical trial, phase iii.pt.

18. clinical trial, phase iv.pt.

19. controlled clinical trial.pt.

20. randomized controlled trial.pt.

21. multicenter study.pt.

22. clinical trial.pt.

23. exp Clinical Trials as Topic/

24. 9 or 10 or 11 or 12 or 13 or 14 or 15 or 16 or 17 or 18 or 19 or 20 or 21 or 22 or 23

25. (clinical adj trial$).tw.

26. ((signl$ or doubl$ or treb$ or tripl$) adj (blind$3 or mask$3)).tw.

27. Placebos/

28. placebo$.tw.

29. randomly allocated.tw.

30. (allocated adj2 random$).tw.

31. 25 or 26 or 27 or 28 or 29 or 30

32. 24 or 31

33. case report.tw.

34. “Abstract report”

35. letter/

36. Historical Article/

37. 33 or 34 or 35

37. 32 not 36

38. 4 and 8 and 37

39. 38

40. limit 38 to yr="2000 -Current"
