# Supplementary material for: The Effectiveness of Therapeutic Exercise Interventions With Virtual Reality on Balance and Walking Among Persons With Chronic Stroke: Systematic Review, Meta-Analysis, and Meta-Regression of Randomized Controlled Trials
Source: J Med Internet Res. 2024 Dec 2;26:e59136. doi: 10.2196/59136 (PMC11650088; doi:10.2196/59136)
Supplement: Multimedia Appendix 2 [file jmir_v26i1e59136_app2.pdf]

# PRISMA 2020 Checklist

| Section and Topic | Item # | Checklist item | Location where item is reported |
|---|---|---|---|
| **TITLE** | | | |
| Title | 1 | Identify the report as a systematic review. | Original file: 59136-899762-1-SM.docx<br>p. 1 |
| **ABSTRACT** | | | |
| Abstract | 2 | See the PRISMA 2020 for Abstracts checklist. | Original file:<br>p. 1 |
| **INTRODUCTION** | | | |
| Rationale | 3 | Describe the rationale for the review in the context of existing knowledge. | Original file<br>p. 2–4/23 |
| Objectives | 4 | Provide an explicit statement of the objective(s) or question(s) the review addresses. | Original file p. 4/23 |
| **METHODS** | | | |
| Eligibility criteria | 5 | Specify the inclusion and exclusion criteria for the review and how studies were grouped for the syntheses. | Original file<br>p. 5/23 |
| Information sources | 6 | Specify all databases, registers, websites, organisations, reference lists and other sources searched or consulted to identify studies. Specify the date when each source was last searched or consulted. | Original file<br>p. 4/23 |
| Search strategy | 7 | Present the full search strategies for all databases, registers and websites, including any filters and limits used. | Original file<br>p. 4/23 and<br>supplementary file 2. |
| Selection process | 8 | Specify the methods used to decide whether a study met the inclusion criteria of the review, including how many reviewers screened each record and each report retrieved, whether they worked independently, and if applicable, details of automation tools used in the process. | Original file<br>p. 6–7/23 |
| Data collection process | 9 | Specify the methods used to collect data from reports, including how many reviewers collected data from each report, whether they worked independently, any processes for obtaining or confirming data from study investigators, and if applicable, details of automation tools used in the process. | Original file<br>p. 6–7/23 |
| Data items | 10a | List and define all outcomes for which data were sought. Specify whether all results that were compatible with each outcome domain in each study were sought (e.g. for all measures, time points, analyses), and if not, the methods used to decide which results to collect. | Original file<br>p. 5/23, figure 1., appendix 7. |
| | 10b | List and define all other variables for which data were sought (e.g. participant and intervention characteristics, funding sources). Describe any assumptions made about any missing or unclear information. | Original file p. 5/23, 9-11/23<br>appendix 4–6 |
| Study risk of bias assessment | 11 | Specify the methods used to assess risk of bias in the included studies, including details of the tool(s) used, how many reviewers assessed each study and whether they worked independently, and if applicable, details of automation tools used in the process. | Original file<br>p. 7/23 and figure 1. |
| Effect measures | 12 | Specify for each outcome the effect measure(s) (e.g. risk ratio, mean difference) used in the synthesis or presentation of results. | Original file<br>p. 7–8/23 |

| Section and Topic | Item # | Checklist item | Location where item is reported |
|---|---|---|---|
| Synthesis methods | 13a | Describe the processes used to decide which studies were eligible for each synthesis (e.g. tabulating the study intervention characteristics and comparing against the planned groups for each synthesis (item #5)). | Original file<br>p. 7–8/23 and Multimedia appendix 1. |
| | 13b | Describe any methods required to prepare the data for presentation or synthesis, such as handling of missing summary statistics, or data conversions. | Original file p. 7/23<br>(additional material on request) |
| | 13c | Describe any methods used to tabulate or visually display results of individual studies and syntheses. | Original file p. 7–9, Figure 2–5 and Multimedia Appendix 2–3. |
| | 13d | Describe any methods used to synthesize results and provide a rationale for the choice(s). If meta-analysis was performed, describe the model(s), method(s) to identify the presence and extent of statistical heterogeneity, and software package(s) used. | Original file<br>p. 7–9/23 |
| | 13e | Describe any methods used to explore possible causes of heterogeneity among study results (e.g. subgroup analysis, meta-regression). | Original file<br>p. 8–9/23 |
| | 13f | Describe any sensitivity analyses conducted to assess robustness of the synthesized results. | Original file p. 8/23 and<br>Multimedia Appendix 2–3. |
| Reporting bias assessment | 14 | Describe any methods used to assess risk of bias due to missing results in a synthesis (arising from reporting biases). | Original file<br>p. 7/23 and Figure 1. |
| Certainty assessment | 15 | Describe any methods used to assess certainty (or confidence) in the body of evidence for an outcome. | Original file<br>p. 9/23 (GRADE) |
| **RESULTS** | | | |
| Study selection | 16a | Describe the results of the search and selection process, from the number of records identified in the search to the number of studies included in the review, ideally using a flow diagram. | Original file<br>p. 9/23 and Multimedia Appendix 1. |
| | 16b | Cite studies that might appear to meet the inclusion criteria, but which were excluded, and explain why they were excluded. | Original file<br>p. 12/23 and Appendix 2 and Multimedia Appendix 1. (flow chart) |
| Study characteristics | 17 | Cite each included study and present its characteristics. | Original file<br>p. 9–11/23, Multimedia Appendix 4–6 |
| Risk of bias in studies | 18 | Present assessments of risk of bias for each included study. | Original file<br>p. 15/23<br>Figure 1. |
| Results of individual studies | 19 | For all outcomes, present, for each study: (a) summary statistics for each group (where appropriate) and (b) an effect estimate and its precision (e.g. confidence/credible interval), ideally using structured tables or plots. | Table 3. and Figure 2–5. |
| Results of syntheses | 20a | For each synthesis, briefly summarise the characteristics and risk of bias among contributing studies. | Original file<br>p. 13–14/23 and Table 4. |

# PRISMA 2020 Checklist

| Section and Topic | Item # | Checklist item | Location where item is reported |
|---|---|---|---|
| | 20b | Present results of all statistical syntheses conducted. If meta-analysis was done, present for each the summary estimate and its precision (e.g. confidence/credible interval) and measures of statistical heterogeneity. If comparing groups, describe the direction of the effect. | Original file p.<br>abstract, 11–14/23, Table 4., Figure 2–5. |
| | 20c | Present results of all investigations of possible causes of heterogeneity among study results. | Original file<br>p. 12–13/23 |
| | 20d | Present results of all sensitivity analyses conducted to assess the robustness of the synthesized results. | Original file p. 12–13, Appendix 2., Appendix 3. |
| Reporting biases | 21 | Present assessments of risk of bias due to missing results (arising from reporting biases) for each synthesis assessed. | Original file<br>p. 12 |
| Certainty of evidence | 22 | Present assessments of certainty (or confidence) in the body of evidence for each outcome assessed. | Original file<br>p. 13–15 |
| **DISCUSSION** | | | |
| Discussion | 23a | Provide a general interpretation of the results in the context of other evidence. | Original file<br>p. 16–18 |
| | 23b | Discuss any limitations of the evidence included in the review. | Original file<br>p. 19–20 |
| | 23c | Discuss any limitations of the review processes used. | Original file<br>p. 19–20 |
| | 23d | Discuss implications of the results for practice, policy, and future research. | Original file p. 20–21 |
| **OTHER INFORMATION** | | | |
| Registration and protocol | 24a | Provide registration information for the review, including register name and registration number, or state that the review was not registered. | Abstract, original file p. 4/23. |
| | 24b | Indicate where the review protocol can be accessed, or state that a protocol was not prepared. | Abstract, original file p. 4/23 and Other file: Refrences: [106] |
| | 24c | Describe and explain any amendments to information provided at registration or in the protocol. | Abstract, original file p. 4/23 and Other file: Refrences: [106] *No changes in the protocol.* |
| Support | 25 | Describe sources of financial or non-financial support for the review, and the role of the funders or sponsors in the review. | Original file<br>p. 22/23 |
| Competing interests | 26 | Declare any competing interests of review authors. | Original file<br>p. 22/23 |
| Availability of data, code and other materials | 27 | Report which of the following are publicly available and where they can be found: template data collection forms; data extracted from included studies; data used for all analyses; analytic code; any other materials used in the review. | https://helda.helsinki.fi/server/api/core/bitstreams/44e412d4-04ea-4b6f-8902-e424401d81eb/content |

*From:* Page MJ, McKenzie JE, Bossuyt PM, Boutron I, Hoffmann TC, Mulrow CD, et al. The PRISMA 2020 statement: an updated guideline for reporting systematic reviews. BMJ 2021;372:n71. doi: 10.1136/bmj.n71
For more information, visit: http://www.prisma-statement.org/
