# Supplementary material for: The Effectiveness of Therapeutic Exercise Interventions With Virtual Reality on Balance and Walking Among Persons With Chronic Stroke: Systematic Review, Meta-Analysis, and Meta-Regression of Randomized Controlled Trials
Source: J Med Internet Res. 2024 Dec 2;26:e59136. doi: 10.2196/59136 (PMC11650088; doi:10.2196/59136)
Supplement: Multimedia Appendix 3 [file jmir_v26i1e59136_app3.docx]

**Priority listing**

Priority of balance and walking outcomes was determined for the standardized mean difference analyses. The prioritization list was defined by the incidence of the measure in the data and the suitability of the outcome for the chronic stroke population according to scientific practice recommendations, which consider the validity, reliability, usability, and sensitivity of the measure to detect the change.

Prioritization of balance and walking outcomes in the meta-analysis.

|  | Meter priority | Target of the measure | ICF component |
| --- | --- | --- | --- |
| Balance outcomes: | 1. Berg’s Balance Scale (BBS) | Changing and maintaining body position | Performance |
|  | 2. Functional Reach Test (FRT) | Changing and maintaining body position | Performance |
|  | 3. Activities-specific Balance Confidence Scale (ABC) | Balance at the level of participation | Capacity |
|  | 4. Timed ”Up & Go” Test (TUG) | Walking balance | Performance |

.

|  | Meter priority | Target of the measure | ICF component |  |
| --- | --- | --- | --- | --- |
| Walking outcomes: | 1. Six minute walk test (6MWT) | Walking long distances | Performance |  |
|  | 2. Two minute walk test (2MWT) | Walking short or long distances | Performance |  |
|  | 3. 10 metre walk test (10mWT) | Walking short distances | Performance |  |
|  | 4. Cadence steps/min (GAITRITE, Smart step) | Walking short distances/gait pattern functions | Performance |  |
|  | ICF; International Classification of Functioning, Disability and Health | | | |
