# Supplementary material for: The Effectiveness of Therapeutic Exercise Interventions With Virtual Reality on Balance and Walking Among Persons With Chronic Stroke: Systematic Review, Meta-Analysis, and Meta-Regression of Randomized Controlled Trials
Source: J Med Internet Res. 2024 Dec 2;26:e59136. doi: 10.2196/59136 (PMC11650088; doi:10.2196/59136)
Supplement: Multimedia Appendix 4 [file jmir_v26i1e59136_app4.docx]

Intervention, comparison, and outcome variables of included studies.

| Study | Inclusion criteria | Exclusion criteria | Intervention (with VR) | Intervention emphasis | Control group or other intervention group to which VR intervention is compared | Type of VR method | Outcome variables accepted in the statistical analysis |
| --- | --- | --- | --- | --- | --- | --- | --- |
| Bang et al [95] | Those who could perform communication, comply with instructions in this study, perform balancing and walking independently, had no pain limiting execution of exercise, and had no disability in sight, hearing, and the vestibular organs were selected to be the subjects of this study. | Not reported. | Wii Balance Board^TM^ | Balance | Treadmill training (self-paced) | CG | Cadence |
| Barcala et al [92] | Weekly physical therapy sessions at the institution, the ability to remain in an orthostatic position without support, absence of osteoarticular deformities, and the ability to understand the visual biofeedback. | Associated diseases not pertinent to the physiopathology of stroke were excluded from the study | Wii Balance Board^TM^, conventional physical therapy | Balance | Conventional physical therapy | CG | BBS, TUG |
| Cho & Lee [58] | Hemiparesis resulting from a single stroke for more than 6 months; able to understand and follow simple verbal instructions (Korean version of Mini-Mental State Examination score >24); Brunnstrom score between 1-4 for the lower extremity; no serious visual impairment or hearing disorder. | Severe heart disease or uncontrolled hypertension and pain; any neurologic or orthopedic disease | Walking training programme using a real-world video recording, a standard rehabilitation programme, FES | Walking | Treadmill training, a standard rehabilitation programme, FES | VE | BBS, TUG, walking velocity, cadence |
| Cho & Lee [59] | Hemiparesis resulting from a single stroke for more than 6 months; ability to walk with and without use of an assistive device for 10 m; able to understand and follow simple verbal instructions (Korean version of Mini-Mental State Examination score >24) and no severe heart disease or uncontrolled hypertension. | Orthopedic and other gait influencing diseases such as arthrosis or total hip joint replacement and participation in other studies or rehabilitation programs. | Treadmill training with real world video recording, a standard rehabilitation programme, FES | Walking | Treadmill gait training, a standard rehabilitation programme, FES | VE | BBS, TUG, walking velocity, cadence |
| Cho et al [60] | Hemiparesis resulting from a single stroke for more than 6 months; ability to walk with and without use of an assistive device for 10 m; able to understand and follow simple verbal instructions (Korean version of Mini-Mental State Examination score >24) and no severe heart disease or uncontrolled hypertension. | Severe dementia or aphasia; hemispatial neglect; ataxia or any other cerebellar symptom; or participation in other studies or rehabilitation programs. | Wii Balance Board^TM^, a standard rehabilitation programme | Balance | A standard rehabilitation programme | CG | BBS, TUG |
| Choi et al [61] | At least a year after first stroke; mini-mental state examination (MMSE) score >24; motor-free visual perception test-3 (MVPT-3) score <45; ability to understand instructions; ability to stand for 30 minutes independently; no spatial neglect. | Prescribed drugs that affect balance; diagnosed with orthopedic diseases (arthritis, fracture, and low back pain); receiving parallel treatments in other medical institutions; cerebellar or vestibular dysfunction; visual problem. | Wii Sports^TM^, conventional therapy | Balance | General balance training, conventional therapy | CG | BBS, TUG, 10mWT |
| Choi et al [93] | Diagnosis > 6 months; ability to stand independently; no cognitive impairment based on Mini-Mental State Examination results. | Neurologic problem apart from stroke; orthopaedic impairment; abnormal senses of vision and/or hearing. | Wii Balance Board^TM^, Constraint-induced movement therapy, traditional physical therapy | Balance | Traditional physical therapy | CG | FRT, TUG |
| Druzbicki et al [94] | History of stroke; time since the onset of stroke < 6 months; ability to walk independently; lower limb motricity 3-5 on the Brunnström scale; disability score between 1-3 on the Rankin scale; spasticity of paretic lower limb not greater than 1+ on the modified Ashworth scale; altered weightbearing symmetry in lower limbs while standing (symmetry index > 1.15) | Disturbed higher mental functions that limited comprehension and ability to fulfil the tasks during the training sessions; visual field defects; concomitant orthopaedic disorders; flexion contracture at the ankle; periodic cardiac or pulmonary insufficiency preventing participations in additional exercise with the treadmill; lack of consent to additional exercise | Treadmill training with biofeedback and real-time visualization, standard rehabilitation programme consisting of NDT, Bobath, PNF | Walking | Standard rehabilitation programme consisting of NDT, Bobath, PNF | RTV | Walking velocity |
| Fishbein et al [89] | Hemiplegia after a stroke for at least 1 year since the incident; age range 40-80 years old; not taking medication or with unchanged medication throughout the past half year or longer; using an ankle-foot orthosis or having no splint at all; and can do a regular walk of 10 m without and with single point stick. | Major cardiac problems; a score of less than 25 in the Mini-Mental test; fractures or severe orthopedic limitations that do not allow for training, and which occurred over the last 6 months; and more than 3 falls in the year prior to participating in the study. | Virtual Reality-based dual task of an upper extremity while treadmill walking | Walking | Single-task treadmill walking | VE | BBS, FRT, TUG, ABC, 10mWT |
| Fritz et al [82] | Clinical presentation of lower extremity  unilateral hemiplegia; more than 6 months post  stroke, ability to stand with minimal physical  assistance without an assistive device for 5  Minutes; and ability to ambulate 10 feet with or  without assistance. | Unable to ambulate 150 feet prior to stroke;  severe hypertension with systolic > 200 mm Hg and diastolic > 110 mm Hg at rest; history of serious chronic obstructive pulmonary disease; severe arthritis or orthopaedic problems or severe weight-bearing pain; lower extremity amputation; nonhealing ulcers on the lower extremity; severe visual or hearing impairment; history of deep venous thrombosis or pulmonary embolism  within 6 months; uncontrollable diabetes; history of severe seizure disorder; other neurological conditions such as multiple sclerosis or Parkinson’s disease; pain scored greater than 5/10 on a visual analog scale; current physical therapy; or health  problems judged by the screening physician to put the client at significant risk of harm during the study. | Wii Balance Board^TM^, Wii Sports^TM^, PlayStation^TM^ EyeToy Play 2, Play Station Kinetic^TM^ training | Balance | No treatment | CG | BBS, TUG, 6MWT (walking velocity) |
| Givon et al [75] | Lived in the community; 18 to 80 years of age and sustained a single stroke at least 6 months prior to the study; ability to walk at least 10-meters (with or without an assistive device  or physical assistance); weakness of the upper  extremity (determined by the upper extremity subtest  of the Fugl-Meyer Motor Assessment); without a significant cognitive deficit (score above 21 points on The Mini Mental State Examination). | Other neurological conditions or  epilepsy | Wii Sports^TM^, SeeMe-system, Microsoft Kinect^TM^ training | Walking | Traditional therapy | CG | 10mWT (walking velocity) |
| Gok et al [83] | First episode of unilateral cerebrovascular accident with hemiplegia; ability to understand and follow commands; ambulatory before stroke; no medical contraindication to exercise; ability to stand without assistance at least for 1 minute. | A history of any other neurological pathology; conditions affecting balance; neglect; dementia; impaired vision or concomitant  medical illness or musculoskeletal conditions  affecting the lower limbs. | Kinaesthetic Ability Training device (KAT), conventional therapy | Balance | Conventional therapy | CG | - |
| Hung et al [88] | Post-stroke duration of at least 6 months; age ≥18 years; ability understand verbal instructions and learn; adequate visual acuity (with appropriate correction, if necessary); and ability to walk independently with or without device. | Bilateral hemispheric or cerebellar lesions; aphasia; significant visual field deficits or hemineglect; or a history of orthopedic or other neurological diseases; and/or medical conditions that would prevent adherence to the exercise protocol. | Wii Sports^TM^ | Balance | Conventional weight-shifting training | CG | BBS |
| Hung et al [96] | Hemiplegic stroke at least 6 months prior to enrollment; were aged > 18 years; had a Berg Balance Scale score <56; were able to understand verbal instructions and watch a television screen satisfactorily; and were able to walk independently with or without device 10m. | Bilateral hemispheric or cerebellar lesions; receptive aphasia; significant visual field deficits or hemineglect; and concomitant other neurologic diagnoses; or conditions that would prevent adherence to the exercise protocol. | Wii Balance Board^TM^ training | Balance | Conventional weight-shift raining | CG | FRT, TUG |
| In et al [76] | Ability to understand and follow simple verbal instructions; had a Mini Mental State Examination (MMSE) score over 21; had a Brunnstrom score between stage I and IV; had no apraxia or hemineglect and; had no orthopedic and neurologic conditions such as fractures and digital neuropathy on their lower extremities. |  | Virtual Reality reflection therapy, conventional therapy | Voluntary muscle activity | Placebo Virtual Reality reflection therapy, conventional therapy | RTV | BBS, FRT, TUG, 10mWT (walking velocity) |
| Jung et al [62] | History of falling; occurrence of first hemiparetic stroke 6 months before the study; ability to walk independently for more than 30 minutes; nocognitive impairment (>24 in a mini-mental state examination); Brunnstorm stage >4 and no cardiovascular, orthopedic, or other neurologic conditions that may interfere with the study. |  | Virtual Reality treadmill training (simulated walks in a park) | Walking | Treadmill training | VE | ABC, TUG |
| Jung et al. 2011 [63] | Those who were diagnosed with hemiplegia due to cerebral infarction or cerebral hemorrhage; whose length of disease was at least 3 months; whose Mini-Mental State Exam-Korea (MMSE-K) score was 24 or higher; who had no problem in looking at monitor; who had no hemineglect; who could walk at least 15m; and who could understand and follow the researcher’s instructions. |  | 3D Thera-Balance training, neurophysiological treatment | Balance | Neurophysiological treatment, weight-shift training | RD | Berg, 10mWT (walking velocity) |
| Kang et al [77] | Hemiparetic stroke patients six months after diagnosis; patients who could walk on their own for more than 15 minutes; patients without visual disabilities or hemianopia; patients who had a mini-mental state examination score of 21 or higher; and Brunnstorm stage > 4. | Cardiovascular problems; orthopaedic and other cardiovascular problems; orthopaedic and other neurological diseases except stroke for influencing gait. | Treadmill training with optic flow (simulated walking in street) | Walking | Conventional physical therapy | VE | FRT, TUG, 6MWT (walking velocity) |
| Kim et al [78] | Presence of hemiparesis secondary to stroke that had occurred less than 2 years but more than 6 months ago; ability to walk 10 m independently with or without an assistive device or person; ability to communicate and understand, as indicated by a Mini-Mental Status Examination score of more than 21 points; and no visual disorders or visual field deficits. | Use of medication or other therapies for cardiovascular disease or metabolic disorder; and known musculoskeletal conditions that would affect the ability to walk safely. | The Biodex Balance System^TM^ training, conventional physical therapy | Balance | Conventional physical therapy | RD | FRT, 10mWT (walking velocity) |
| Kim, H. et al [64] | Hemiplegic subjects who had experienced a middle cerebral lesion and stroke at least 6 months earlier to exclude natural restoration; subjects who scored greater than 24 points on the Mini-Mental State Examination-Korea; and could understand and follow the intervention and walk 10 m independently. | Vestibular system disease: cerebellar disease, visual disability; visudal defect, sensory aphasia; auditory disability; auditory defect, neurologic diseases that could have affected the study. | VT-dual-task training on the treadmill, traditional physical therapy | Walking | Treadmill training, traditional physical therapy | VE | Step length, cadence |
| Kim, N. et al [65] | History of stroke onset of >6 months prior the study, to minimize the effects of natural recovery; ability to walk without using a walking aid for a minimum of 15 m; Mini-Mental State Examination score of >24 out of 30; and ability to comprehend and follow simple instructions. | Neurological condition; orthopedic disease; or visual impairment. | Treadmill training with VR video, conventional physical therapy | Walking | Conventional physical therapy | VE | - |
| Kim et al [91] | Patients who had experienced the first episode of unilateral stroke with hemiparesis during the previous 6 months; and were capable of comprehending commands; and walking for at least 6 min with or without the use of an assistive device; had no previous history of cardiovascular or respiratory problems; were receiving no medications that would influence the metabolic or cardiorespiratory responses to exercise; had no history of regular exercise training or sports activity to strengthen ventilator muscles; and had no bone deformities of the chest or spine. |  | A respiratory exercise device (game play), an automated full body workout, basic exercise treatment | Respiratory muscle training | An automated full body workout, basic exercise treatment | RD | 6MWT (walking velocity) |
| Kim et al [79] | Inpatients who were diagnosed with stroke by magnetic resonance imaging (MRI) or Computed Tomography (CT) more than 6 months earlier; All the subjects scored 19 or higher points on the mini-mental status examination (MMSE); and were able to maintain an upright posture without any assistance. | Those who had received orthopedic surgery; or had a history of arthritis; hand or upper limb pain; epilepsy; or psychiatric illnesses; Those who had played a virtual reality game before. | Interactive gameplay with Nintendo Wii^TM^, general exercise, electrical stimulation to tibialis anterior | Vertical training | General exercise, electrical stimulation to tibialis anterior | CG | - |
| Kim & Lee [66] | Stroke with onset of six months or longer; independent gait; and Mini-Mental State Examination (MMSE) scores of 24 or higher. |  | VR based FES, treadmill training, general physical therapy | Walking | Treadmill training, general physical therapy | VR | BBS |
| Kim et al [84] | ≥1 yr after first stroke; plateau in the maximum motor recovery after a conventional neurorehabilitation; and the ability to stand for 30 mins; and walk indoors independently (~30-m distance). | Severe visual (i.e., visual neglect determined by the motor free visual perception test); and cognitive impairments; and musculoskeletal disorders that could potentially interfere with experimental tests. | The IREX VR system, conventional physical therapy | Balance and stepping exercise | Conventional physical therapy | RD | BBS, 10mWT (walking velocity, cadence) |
| Lee & Bae [67] | ≥6 months since the diagnosis of stroke; independent gait without device; a trunk umpairment scale (TISall) score of ≥10, a Mini-Mental State Examination (MMSE) score of >24; unimpaired visual and hearing functions; and ability to understand and follow the experimental protocol. | Pre-existing neurological disorder; those who had a progressive disease; or other concurrent medical conditions. | Conventional physical therapy, driving based interactive video play | Task oriented training | Conventional physical therapy,treadmill training | CG | TUG |
| Lee et al [85] | Age between 20 and 75 years; having chronicity >6 months; ability to understand game instructions; ability to stand for 15 minutes; and having Brunnstorm stage ≥3. | Having a Montreal Cognitive Assessment score <16; having visual or auditory impairment with the ability to clearly see or hear feedback from the game; having severe spasticity of lower extremity (Modified Ashworth Scale ≥3; and having other medical symptoms that could affect movement. | Microsoft Kinect^TM^, standard rehabilitation | Balance | Standard rehabilitation | CG | BBS, FRT, ABC |
| Lee, H. et al [68] | Stroke of >6 months duration; a score of >24 points on the Korean versionof the Mini-Mental State Examination; ability to walk a distance of 10 m with or without an auxiliary device; no history of orthopedic conditions involving the lower limbs; ability to follow instructions and perform the exercise programs; and no visual or hearing impairment. |  | Wii Sports^TM^, Wii Balance Board^TM^, conventional physical training | Balance | Task-oriented training, conventional physical training | CG | FRT |
| Lee, I-W et al [69] | Over 24 points of the Mini-Mental State Examination (MMSE); could independently walk over 10 meters; could acknowledge understanding of instructions and communication; had no visual impairment; visual field of defects; or orthopedic disease. |  | Virtual reality exercise programme using BioRescue, cognitive tasks | Balance and stepping exercise | PNF training | RD | BBS, TUG |
| Lee et al [49] | A diagnosis of stroke for at least 6 months (chronic stroke); not taking medication that can affect balance; Mini-Mental State Examination (MMSE) score <24; no pain or disability associated with acute musculoskeletal conditions; sitting to sidelying with moderate assistance; sitting for longer than 10 seconds without support; and standing without support for 1 minute. | Pusher syndrome. | An Super Video Graphics Array (SVGA), head-mounted display (HMD), a general physical therapy programme | Postural control training | A general physical therapy programme | VE | BBS, TUG, step length, cadence |
| Lee et al [90] | The patients at six months or greater after the  Stoke; the first episode of unilateral stroke (infarction, hemorrhage) with hemiparesis, in the territory of the  internal carotid artery; the diagnosis of stroke con-  firmed by computed tomography or magnetic resonance  Imaging; the ability to understand and follow simple  verbal instructions; was ambulatory before onset of  Stroke; the ability to walk 10 meters independently | They were excluded if they were medically unstable or had a history of musculoskeletal conditions affecting lower limbs or neurological diseases affecting vision, gait balance, conscious or cognitive level (Mini-Mental State Examination [MMSE] <24). The protocol was approved by the Institutional Review Board (IRB) and informed consent was obtained prior to training. | Balance Control Trainer (BCT), conventional physical therapy | Balance | Conventional physical therapy | RD | BBS, TUG, 10mWT (walking velocity) |
| Lin et al [87] | A history of cerebral vascular accident (including first and recurrent stroke) for more than six months; living in long-term care facilities (LTCFs) for more than three months; having active movement of the proximal part of upper extremity in the hemiparetic side (Brunnstrom stage U/E ≥ 3); being able to sit for short periods without hand support for at least 30 s; having cognitive status screened using the Mini-Cog test and being able to follow the instruction; and; being able to communicate and follow a three-step command. | Having other neuromusculoskeletal condition and systemic diseases, such as Parkinson’s disease and uncontrolled heart disease; blindness and deafness; having a psychiatric history. | A WSN telerehabilitation system: 3D-animated practice videos, interactive videogames | Balance | Conventional balance training | CG/RD | BBS |
| Lloréns et al [86] | Hemiparesia; age ≥40 years old and ≤70 years old; chronicity >6 months; absence of cognitive impairment (Mini-Mental State Examination 12 cut-off >); able to follow instructions; ability to maintain stride-standing position for 30 seconds without holding onto or assistance from another person as specified in the Brunel Balance Assessment, section 3, level 7. | Individuals with severe dementia or aphasia  (Mississippi Aphasia Screening Test 14 cut-off <45); Individuals whose visual or hearing impairment  did not allow the possibility of interaction with the  system; Individuals with hemispatial neglect; Individuals with ataxia or any other cerebellar symptom. | Virtual reality-based training, conventional training | Balance/stepping exercise | Conventional therapy | N/A | BBS, 10mWT (walking velocity) |
| Lloréns et al [80] | Age 40 and 75 years; chronicity >6 months; Brunel Balance Assessment (BBA) 19: section 3, levels  7 to 12; Mini-Mental State Examination score 20>23;  Internet access in their homes. | Individuals with severe aphasia (Mississippi Aphasia Screening Test 21 cutoff score <45); individuals with hemispatial neglect; individuals with ataxia or any other cerebellar symptom. | VR-telerehabilitation in home environment | Balance | VR-telerehabilitation in clinic | N/A | - |
| Park et al [81] | A period of more than 6 months  between stroke and randomization; hemiplegic stroke as  diagnosed by a neurologist, a total score of 21 or greater  on the Mini–Mental State Examination (MMSE); no problems with auditory or visual functioning, an ability to walk more than 10 m with or without assistive devices;  not taking any medication that could inﬂuence balance;  stable vital signs, a capacity to provide informed  consent. | Severe conditions that require medical care, such as uncontrolled blood pressure or angina; musculoskeletal impairments of the lower extremity; psychological conditions; or the refusal to use a video game. | Xbox Kinect^TM^ games | Balance | Conventional physical therapy | CG | BBS, TUG, 10mWT |
| Park et al [70] | A diagnosis of stroke at least six months prior to the start of the study; ability to walk independently without use of a walking aids for more than 10 meters; able to understand and follow simple verbal in-  structions (The Korean version of the Mini-Mental State Examination score greater than 24 of 30); did not have a serious visual impairment or hearing disorder. | Patients with any comorbidity or  neurologic or orthopedic disease that could potentially interfere with the study; language difficulty that would affect information reception. | Virtual reality-based postural control training, conventional physical therapy | Postural control training | Conventional physical therapy | VE | 10mWT (walking velocity) cadence, step length |
| Pedreira da Fonseca et al [97] | Patients of both sexes, with hemiparesis after a stroke;  the age group from 18 to 65 years. | Injury occurred fewer than 6 months  previously, considering the expected time for spontaneous recovery; patients with associated disorders such as epilepsy, and sensory and perceptual deﬁcits such as hemineglect and Pusher syndrome; patients with osteodegenerative disorders that would prevent participation in the games or that could inﬂuence the body balance; individuals who had cognitive and communication disorders, affecting understanding, that could compromise performance in the games. | Nintendo Wii^TM^, conventional physical therapy | Dynamic balance | Conventional therapy | CG | - |
| Shin & Song [71] | Chronic hemiplegia for more than 6 months resulting  from a single stroke; the ability to sit independently  for at least 30 minutes; the ability to walk with or  without the use of an assistive device for 10 minutes;  the ability to understand and follow simple verbal in-  structions (Korean version of Mini-Mental State Ex-  amination score 924). ) | The exclusion criteria included participation in other studies or rehabilitation programs, orthopedic or other conditions or diseases that inﬂuence balance and gait such as arthritis or total hip joint replacement, use of balance-inﬂuencing drugs such as opiates or antibiotic streptomycin, severe defects in vision, and visual perception deﬁcits that may affect the visual feedback trunk control training (Motor-Free Visual Perception Test score G20). | Smartphone-Based Visual Feedback Trunk Control Training (SPVFTCT) + conventional rehabilitation | Trunk control training | Conventional rehabilitation | RD | TUG |
| Song & Park [72] | No visual field defect; no abnormality in the vestibular organs; no orthopedic diseace; an unrestricted range of motion; ability to understand and performthe exercise as  instructed by the researchers; a score of 24 or higher on the Mini-Mental State Examination-Korea version. | Not reported. | Kinect Xbox^TM^ | Balance/stepping exercise | MOTOMed Viva 2 ergometer training | CG | TUG, 10mWT (walking velocity) |
| Yang et al [99] | Hemiparetic from a single stroke occur-  ring at least 6 months earlier; limited household walker,  unlimited household walker, or most-limited community walker by functional walking category; not presently receiving any rehabilitation services; no visual ﬁeld deﬁcit or hemianopia; stable medical condition to allow participation in the testing protocol and intervention; an ability to understand instructions and follow commands. | Patient with any comorbidity or disability other than stroke that would preclude gait training; any uncontrolled health condition for which exercise is contraindicated; any neurological or orthopedic diseases that might interfere with the study. | Virtual reality-based treadmill training | Walking | Treadmill training | VE | ABC, 10mWT (walking velocity) |
| Yatar & Yildirim [98] | The first ever stroke with hemiparesis (≥ 6 months). The criterion for inclusion was first  ever stroke with hemiparesis (≥ 6 months). | If they had any other physical problem or epilepsy;  moderate to severe cognitive deficits as evaluated by the Mini Mental State Examination test (MMSE) (≤20 points); severe depression as measured by the Beck Depression Inventory (BDI) (≥30 points); were unable to walk independently according to the Modified Rankin Scale (MRS) (> 3 points). | Wii Fit^TM^, progressive balance training, exercise based on NDT | Balance | Progressive balance training, exercise based on NDT | CG | BBS, FRT, TUG |
| Yom et al [73] | Hemiparesis due to stroke with onset of >6  Months; the ability to follow verbal instructions, the ability to communicate at a certain level, and a score of >24 points on the; Mini-Mental State Examination-Korean (MMSE-K). | If they had diplegia or visual or vestibular impairments. | Virtual reality-based ankle exercise | Balance | Watching a video | VE | TUG, walking velocity, cadence, step length |
| Yu & Cho [74] | Hemiparesis resulting from a single stroke for more than  6 months; able to understand and follow simple verbal instruction (Korean version of Mini-Mental State examination score >24); the absence of a serious visual impairment or hearing disorder; no severe heart disease or uncontrolled hypertension, | Orthopedic and other balance control influencing disease, such as ataxia or any other cerebellar symptoms; participation in other studies or rehabilitation programs. | Standard rehabilitation program, Wii Fit^TM^ balance training | Balance | Standard rehabilitation program | CG | BBS, TUG |
| CG = commercial games, RD = rehabilitation device, VR = virtual reality, VE = virtual environment, RTV = real time visualization. | | | | | | |  |
