# Supplementary material for: The Effectiveness of Therapeutic Exercise Interventions With Virtual Reality on Balance and Walking Among Persons With Chronic Stroke: Systematic Review, Meta-Analysis, and Meta-Regression of Randomized Controlled Trials
Source: J Med Internet Res. 2024 Dec 2;26:e59136. doi: 10.2196/59136 (PMC11650088; doi:10.2196/59136)
Supplement: Multimedia Appendix 5 [file jmir_v26i1e59136_app5.docx]

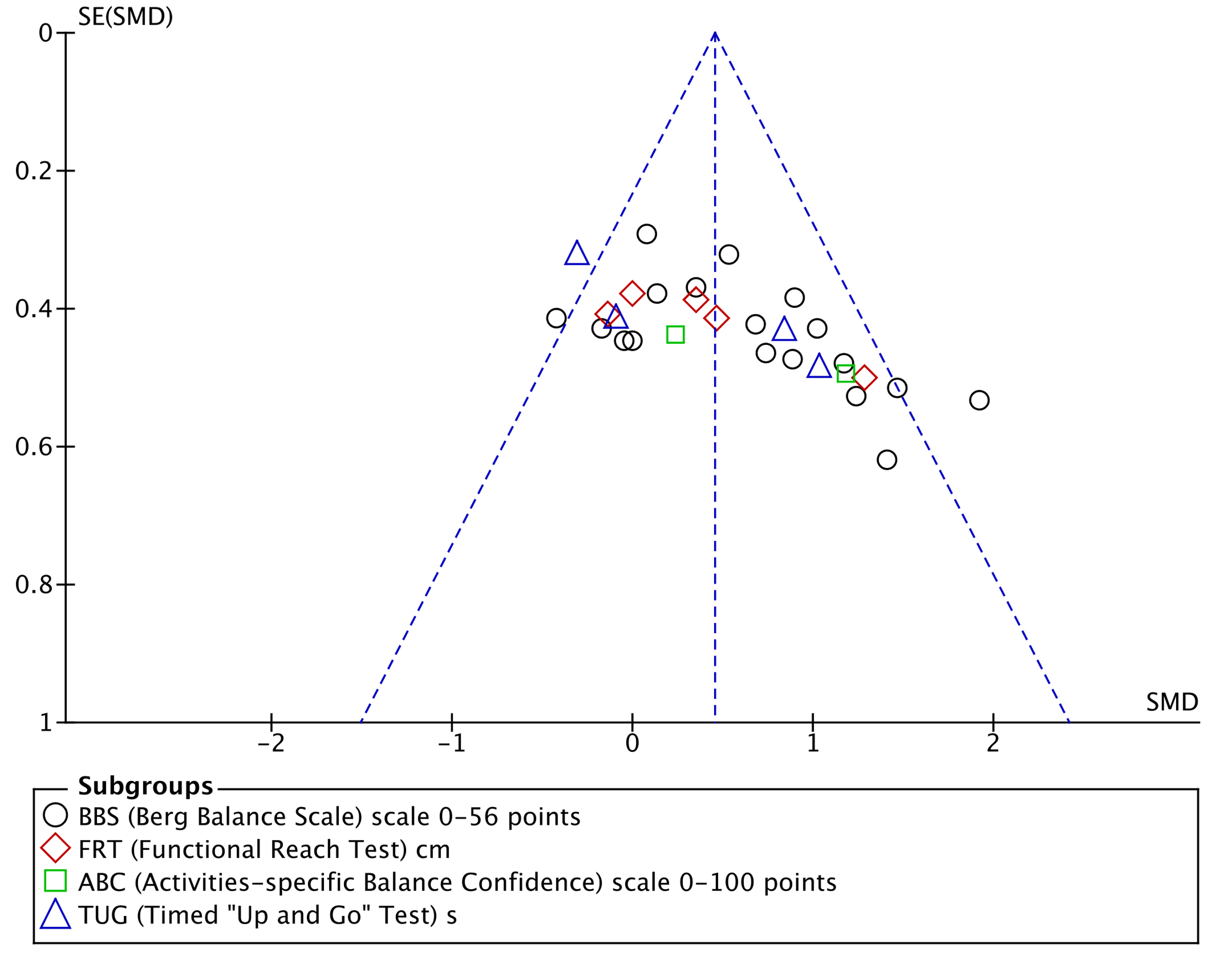


Funnel plot of SMD analysis with balance outcomes.


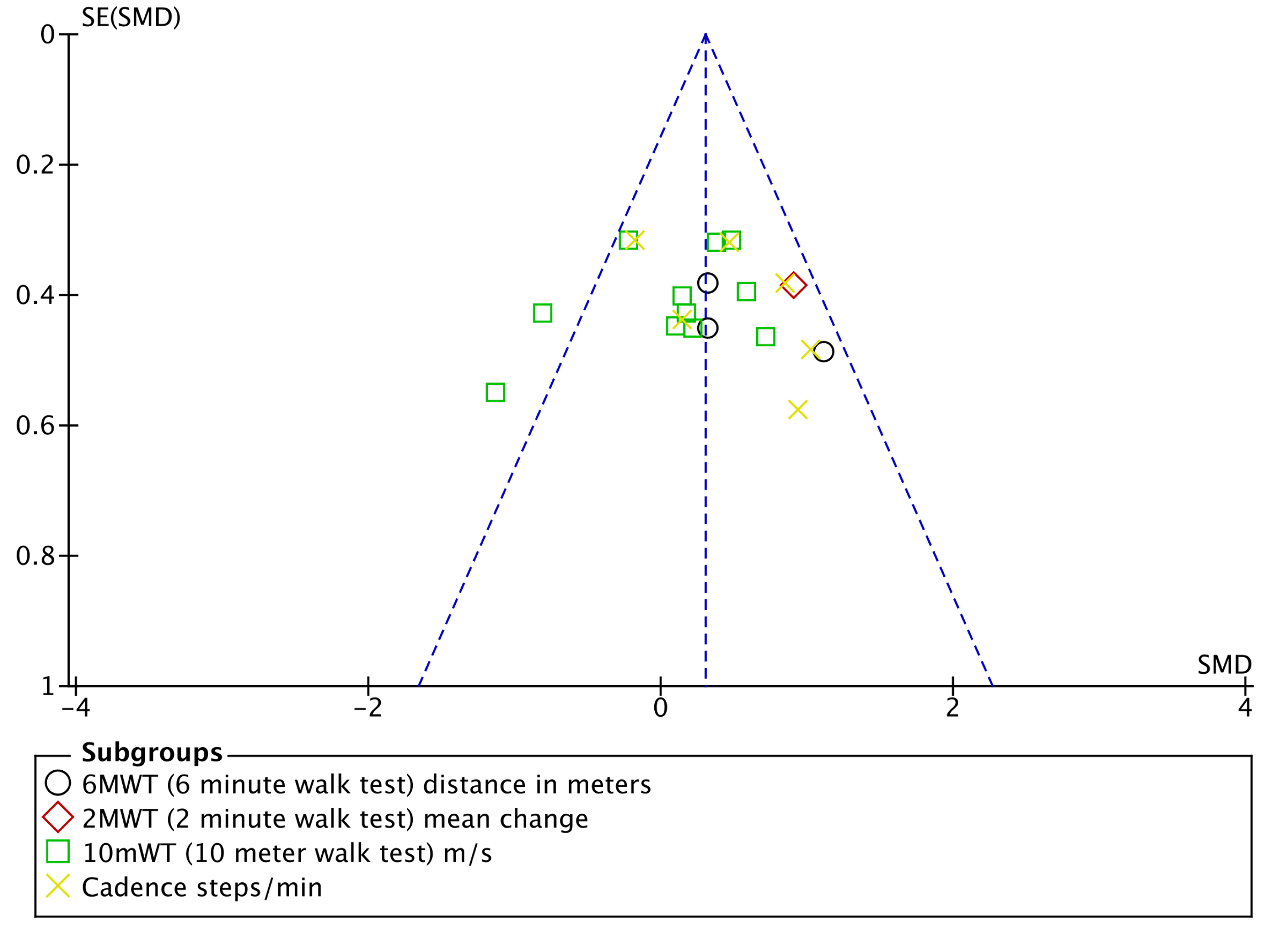


Funnel plot of the SMD analysis with walking outcomes.
