# Supplementary material for: The Effectiveness of Therapeutic Exercise Interventions With Virtual Reality on Balance and Walking Among Persons With Chronic Stroke: Systematic Review, Meta-Analysis, and Meta-Regression of Randomized Controlled Trials
Source: J Med Internet Res. 2024 Dec 2;26:e59136. doi: 10.2196/59136 (PMC11650088; doi:10.2196/59136)

Sensitivity test for standardized mean difference (SMD) of the effectiveness of therapeutic exercise interventions on walking utilizing VR technology.


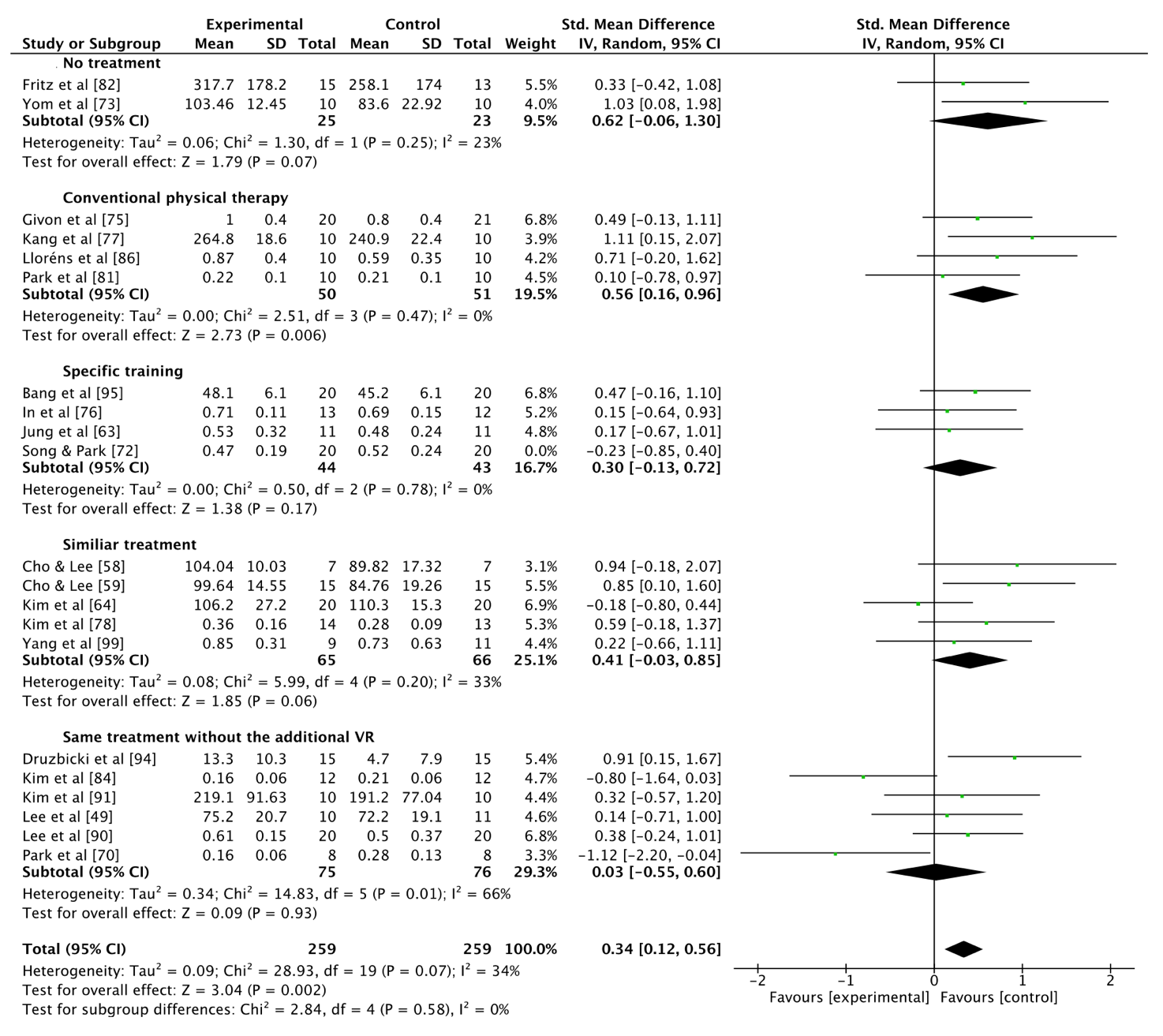

Supplement: Multimedia Appendix 7 [file jmir_v26i1e59136_app7.docx]
